# Endoplasmic reticulum stress reduces caspofungin resistance in *Cryptococcus neoformans*

**DOI:** 10.1128/msphere.00451-26

**Published:** 2026-09-04

**Authors:** Xianya Qu, Guanggan Hu, Christopher W. J. Lee, Linda Horianopoulos, James W. Kronstad

**Affiliations:** 1 The Michael Smith Laboratories, Department of Microbiology and Immunology, University of British Columbia 8166 , Vancouver, British Columbia, Canada; University of Michigan Medical School, Ann Arbor, Michigan, USA

**Keywords:** antifungal, endoplasmic reticulum (ER) stress, caspofungin tolerance, tunicamycin

## Abstract

Echinocandin antifungal drugs, such as caspofungin, inhibit β-1,3-glucan synthesis to impair cell wall formation. Caspofungin is effective in the clinic against *Aspergillus* and *Candida* species but ineffective against *Cryptococcus neoformans*, the fungus responsible for life-threatening meningoencephalitis in immunocompromised individuals. The mechanisms of caspofungin resistance in *C. neoformans* are associated with calcineurin signaling and functions that influence membrane and cell wall composition. The synergistic effects of caspofungin have been studied in combination with various inhibitors, but it is not known whether resistance can be overcome by agents that target the endoplasmic reticulum to influence protein glycosylation, as well as cell wall and membrane composition. Additionally, the role of the polysaccharide capsule of *C. neoformans* in blocking the access of caspofungin to the target Fks1 glucan synthase is not clear. Here, we identified a potential synergistic effect between caspofungin and the glycosylation inhibitor tunicamycin, with a significant reduction in proliferation, capsule attachment, and chitin and β-1,3-glucan levels when the drugs were used in combination. A targeted screen also identified other agents that influence ER function and that potentially display synergy with caspofungin. Overall, our analysis provides evidence that targeting the ER to impair cell wall and membrane integrity and capsule formation is an effective strategy to overcome caspofungin resistance in *C. neoformans*.

**IMPORTANCE:**

*Cryptococcus neoformans* is the major cause of life-threatening meningoencephalitis in immunocompromised people. Unfortunately, the number of antifungal drugs available to treat cryptococcal disease is quite limited, and unlike other pathogenic fungi, *C. neoformans* is intrinsically resistant to the echinocandin group of drugs. These drugs target cell wall biosynthesis, and the mechanisms of cryptococcal resistance are not entirely clear. In this study, we demonstrate synergy between the echinocandin drug caspofungin and tunicamycin, an inhibitor of protein glycosylation in the endoplasmic reticulum. Combined treatment impacts cell wall and plasma membrane composition, thus providing insights into approaches to overcome resistance.

## INTRODUCTION

Diseases caused by fungi are a global threat to human health (1). Echinocandins, such as caspofungin, micafungin, and anidulafungin, are clinically approved antifungal drugs that target synthesis of the fungal cell wall (2). These drugs are widely used to treat invasive fungal diseases such as candidiasis, candidemia, and aspergillosis (3–6). Echinocandins inhibit the synthesis of β-1,3-glucan by the Fks1 glucan synthase, leading to altered cell wall structure, growth inhibition, and death due to osmotic sensitivity (4, 7–9). Echinocandins are ineffective against *Cryptococcus neoformans* due to intrinsic resistance (3, 6, 10). This resistance is unfortunate because *C. neoformans* causes life-threatening meningoencephalitis in immunocompromised people, such as those living with HIV/acquired immunodeficiency syndrome (AIDS) or receiving immunosuppressive therapy (11–13). For example, it is estimated that cryptococcal meningoencephalitis accounts for ~19% of the deaths among people with HIV/AIDS (12). The 1,3-β-glucan synthase of *C. neoformans* is sensitive to echinocandins *in vitro,* and high concentrations will inhibit growth in culture, but the concentrations required to achieve antifungal efficacy are too high to be clinically applicable (6, 8, 10). This inherent resistance limits the use of echinocandins in the treatment of cryptococcosis and highlights the need for alternative therapeutic strategies.

The mechanisms of *C. neoformans* resistance to echinocandins have been explored in a number of studies (14–17). For example, structural aspects of the *C. neoformans* cell surface may influence resistance, including differences in cell wall composition. That is, β-1,3-glucan is the most abundant component in other yeasts, while β-1,6-glucan is the most abundant component in the cell wall of *C. neoformans*, potentially making it less sensitive (7, 18, 19). However, treating *C. neoformans* with caspofungin led to a reduction in both β-1,3-glucans and β-1,6-glucans, and blocking β-1,3-glucan synthesis alters cellular morphology (20, 21). The polysaccharide capsule in *C. neoformans* may also act as a physical barrier to reduce the access of caspofungin to the target glucan synthase (7, 22, 23).

A number of mutant screens have been performed to identify sensitive strains and gain insights into mechanisms of resistance. For example, a screen of insertion mutants on caspofungin revealed sensitivity due to mutations in ergosterol biosynthetic functions and loss of the *CDC50* gene encoding the non-catalytic, regulatory subunit of P4-ATPase phospholipid flippases (24). Another screen of the *C. neoformans* deletion collection revealed a role for calcineurin in caspofungin resistance and identified a number of other functions including a palmitoyltransferase, Ras signaling pathway components, and a chitin synthase that implicated cell wall synthesis (17). A more recent screen identified additional mutants with increased sensitivity to caspofungin, and these had defects in an iron permease, a sterol desaturase, the myosin heavy chain, and a sterol transporter (16). These observations and additional experiments support a key role for membrane integrity in the sensitivity to caspofungin.

Insights from the mutant screens and additional chemical genetic approaches have explored synergistic and additive effects of other drugs and agents with echinocandins for inhibiting *C. neoformans*. In the context of membrane integrity, sodium dodecyl sulfate (SDS), the calcineurin inhibitor FK506, and amphotericin B show synergistic effects with echinocandins (14–16, 25). Additionally, cell wall-disrupting agents, such as photodynamic inactivation with polyethyleneimine-chlorin(e6) (PEI-ce6) (26), and the calcium channel inhibitor verapamil (27) also work synergistically with caspofungin by compromising cell wall integrity and increasing susceptibility. Notably, caspofungin-induced alterations in fungal cell wall structure influence interactions with macrophages for *Candida* species (28) and inflammatory responses to *Aspergillus fumigatus* (29). Furthermore, manumycin A (a protein-farnesyltransferase inhibitor) (30), clorgyline (an efflux inhibitor) (31), 2-bromopalmitate (2BP) (an inhibitor of palmitoyltransferases) (32), and brilacidin (a host defense peptide mimetic) (33) have shown synergistic effects with echinocandins against *C. neoformans* (17). Recently, a screen of natural products identified butyrolactol A as a compound that restores caspofungin sensitivity to the fungus. Subsequent analyses revealed that butyrolactol A inhibits the Apt1-Cdc50 phospholipid flippase to influence phospholipid transport (34). This finding further reinforces the connection between caspofungin resistance and membrane integrity in *C. neoformans*.

In this study, we tested potential synergy between caspofungin and tunicamycin, which inhibits the first step of N-linked glycosylation and induces endoplasmic reticulum (ER) stress to trigger the unfolded protein response (UPR) (35–37). We observed that combined treatment reduced capsule size and impaired capsule attachment, leading to increased capsule shedding. These changes were associated with disruption of the cell wall and a compensatory response that decreased levels of β-glucan and chitin, and increased cell membrane permeability. Functionally, these structural changes correlated with reduced macrophage phagocytosis and decreased intracellular survival of *C. neoformans* after combined treatment. These findings indicated that ER stress influences the sensitivity of *C. neoformans* to caspofungin by disrupting plasma membrane and cell wall integrity. The results for tunicamycin prompted a screen of a library of ER stress-inducing compounds, leading to additional compounds that enhanced caspofungin sensitivity. Overall, these findings reveal a link between ER homeostasis and antifungal efficacy of cell wall-targeting agents, highlighting the ER stress pathway as a potential target for combination therapy against *C. neoformans*.

## RESULTS

### Tunicamycin and caspofungin potentially act synergistically to inhibit growth

Tunicamycin targets the enzyme GlcNAc-1-phosphate transferase to inhibit protein glycosylation, thereby inducing endoplasmic reticulum (ER) stress and activating the unfolded protein response (UPR). We hypothesized that these cellular responses would impact the membrane integrity associated with caspofungin resistance in *C. neoformans* (16, 24, 34, 38). We evaluated potential synergy with two approaches using MIC_50_ and MIC_90_ values and two interpretations of the fractional inhibitory concentration (FIC) index employed for *C. neoformans* (Tables 1 and 2) (14–17, 25, 27, 34, 39). Initial MIC assays were performed using caspofungin concentrations ranging from 0.39 to 200 µg/mL and tunicamycin concentrations from 7.8 to 4,000 ng/mL. We found that the MIC_50_ of tunicamycin was 500 ng/mL at both 30°C and 37°C, while the MIC_50_ for caspofungin was 20 µg/mL at 30°C and 15 µg/mL at 37°C. To determine the FIC value, we narrowed the caspofungin range to 0.31  to 25 µg/mL and the tunicamycin range to 3.5 to 500 ng/mL. In the presence of 125 ng/mL tunicamycin, the MIC_₅₀_ of caspofungin was reduced to 10 µg/mL at 30°C. This effect was more pronounced at 37°C such that only 0.625 µg/mL caspofungin was required to achieve 50% growth inhibition in combination with tunicamycin (Table 1; Fig. S1 and S2). Using the criteria of FIC <1.0 as synergy, =1.0 as additivity, and 1.0–2.0 as autonomous (15, 17, 39), synergy was predicted for the combination at 30°C and 37°C with FIC values of 0.75 and 0.29, respectively (Table 1). The latter value also indicated synergy using the other commonly employed criteria of FIC ≤0.5 as synergy, >0.5–4.0 as indifference, and >4.0 as antagonistic (14, 16, 25, 27, 34). Using MIC_90_ values, the FIC values were 0.75 at both 30°C and 37°C, indicating synergy by only one of the two evaluation approaches (Table 2). We also examined micafungin, another echinocandin antifungal that targets 1,3-β-glucan synthase and which is also approved for the treatment of *Candida* and *Aspergillus* infections (6). We identified the MIC_50_ for micafungin as 64 μg/mL alone and 12.5 μg/mL when combined with tunicamycin. The MIC_50_ for tunicamycin was 500 ng/mL alone and 125 ng/mL when combined with micafungin. When combined with tunicamycin, micafungin exhibited an FIC index of 0.45 for the MIC_50_ values (30°C) (Fig. S3). Based on the above findings, we used 10 µg/mL caspofungin and 125 ng/mL tunicamycin as the treatment concentrations for subsequent experiments.

**TABLE 1:** Minimal inhibitory concentration (MIC_50_) and fractional inhibitory concentration (FIC) index of caspofungin with tunicamycin for strain H99

Temp	MIC_50_				FIC^*a*^	Relationship(s)
	Alone		Combined			
	Tunicamycin	Caspofungin	Tunicamycin	Caspofungin		
30°C	500 ng/mL	20 μg/mL	125 ng/mL	10 μg/mL	0.75	Synergy,^*a*^ indifference^*b*^
37°C	500 ng/mL	15 μg/mL	125 ng/mL	0.625 μg/mL	0.29	Synergy^*a*^^*,^b^*^

^*a*^ FICI <1.0 as synergy, =1.0 as additivity, and 1.0–2.0 as autonomous (15, 17, 39).

^*b*^ FICI ≤0.5 as synergy, >0.5–4.0 as indifference, and >4.0 as antagonistic (14, 16, 25, 27, 34).

**TABLE 2:** Minimal inhibitory concentration (MIC_90_) and fractional inhibitory concentration (FIC) index of caspofungin with tunicamycin for strain H99

Temp	MIC_90_				FIC	Relationships
	Alone		Combined			
	Tunicamycin	Caspofungin	Tunicamycin	Caspofungin		
30°C	500 ng/mL	25 μg/mL	125 ng/mL	12.5 μg/mL	0.75	Synergy,^*a*^ indifference^*b*^
37°C	500 ng/mL	20 μg/mL	125 ng/mL	10 μg/mL	0.75	Synergy,^*a*^ indifference^*b*^

^*a*^ FICI <1.0 as synergy, =1.0 as additivity, and 1.0–2.0 as autonomous (15, 17, 39).

^*b*^ FICI ≤0.5 as synergy, >0.5–4.0 as indifference, and >4.0 as antagonistic (14, 16, 25, 27, 34).

### The combination of caspofungin and tunicamycin increases capsule shedding

We previously demonstrated that tunicamycin inhibits capsule formation in *C. neoformans* (40). We confirmed this observation and found that a combined treatment with tunicamycin and caspofungin further reduced capsule size compared with either drug alone (Fig. 1A and B). *C. neoformans* mutants such as *cap59*Δ*, pka1*Δ*,* or *lac1*Δ, which lack capsule and/or melanin, remain resistant to caspofungin at levels comparable to the WT strain, suggesting that capsule interference with access may not be the only reason for echinocandin resistance (24). We therefore examined capsule shedding for cells grown with the drug combinations, and used the same samples for measurements of cell-associated capsule. We found increased shedding of capsule material in the supernatant after treatment with both caspofungin and tunicamycin, with a greater effect at 37°C (Fig. 1C). This result indicated impaired capsule attachment to the cell surface, potentially due to alterations in cell wall and plasma membrane structure. Notably, impaired capsule attachment was not observed in cells treated with caspofungin alone, further supporting a potentially synergistic effect with tunicamycin. We note that phenotypic tests that compare treatments with single drugs at sub-inhibitory levels for growth with the inhibitory combination of caspofungin and tunicamycin may not give a complete picture because of possible confounding differences in stress levels for the treated cells. Additional phenotypic comparisons could be made between the impact of the combined versus individual drugs at different concentrations closer to the MIC_50_ values of each drug alone.

**Fig 1:**
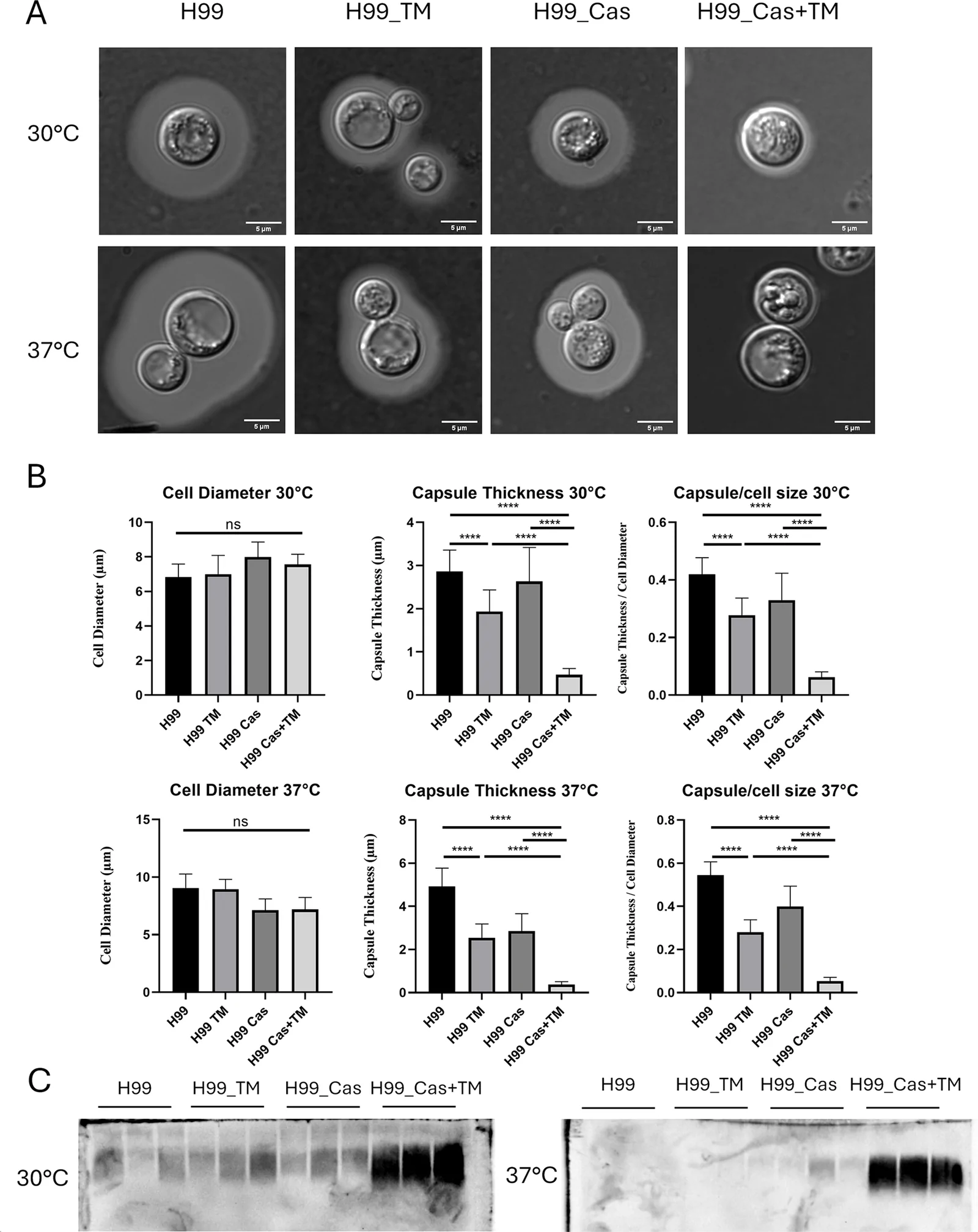
Potential synergistic effects of caspofungin and tunicamycin: Capsule suppression and enhanced polysaccharide shedding. (**A**) Differential interference contrast microscopy (DIC) of capsule stained with India ink for the WT strain (H99) grown at 30°C or 37°C in LIM for 48 h with or without drug treatment: fresh LIM, LIM with 10 µg/mL caspofungin (CAS), LIM with 125 ng/mL tunicamycin (TM), or LIM containing both caspofungin and tunicamycin (scale bar = 5 µm). Note that the representative H99 cell with CAS treatment was also part of the experiment shown in Fig. 8A. (**B**) Measurement of cell size, capsule thickness, and ratio of capsule/cell size from panel **A** (*n* = 100). Three independent experiments were conducted, and the mean and standard deviation of measurements are shown. (**C**) Immunoblot of shed capsule polysaccharide in culture supernatants after 48 h of growth in LIM. Three biological replicates are shown, and the assays were repeated three times. Statistical significance was determined by performing one-way ANOVA and Tukey’s multiple comparisons in GraphPad Prism 8.0 (GraphPad Software) (ns, not significant; ****P < 0.001).

### Caspofungin and tunicamycin alter cell wall composition and membrane integrity

Caspofungin treatment leads to a decrease in both β-1,3-glucans and β-1,6-glucans but an increase in chitin and chitosan levels in *C. neoformans* and *Candida* species (17, 20, 28, 41, 42). We therefore next examined changes in cell wall structure upon combined treatment with caspofungin and tunicamycin. We used flow cytometry after staining with calcofluor white (CFW), wheat germ agglutinin (WGA), or aniline blue to evaluate the levels of chitin, N-acetylglucosamine (GlcNAc), and β-1,3-glucan, respectively. We found that caspofungin treatment at 30°C resulted in increased chitin levels but decreased β-1,3-glucan levels; these changes were not observed at 37°C (Fig. 2A). However, when caspofungin was combined with tunicamycin, both chitin and β-glucan levels were reduced, indicating altered cell wall composition upon treatment with both drugs (Fig. 2A). A previous study similarly reported that the combination of the chitin synthase inhibitor nikkomycin Z and caspofungin led to reduced exposure of both chitin and β-1,3-glucan in *Candida albicans* (43). To eliminate the influence of the capsule on staining of cell wall components, we used the acapsular strains *cap60*Δ and *cap59*Δ and found similar results to the WT strain upon treatment with caspofungin and tunicamycin (Fig. S4).

**Fig 2:**
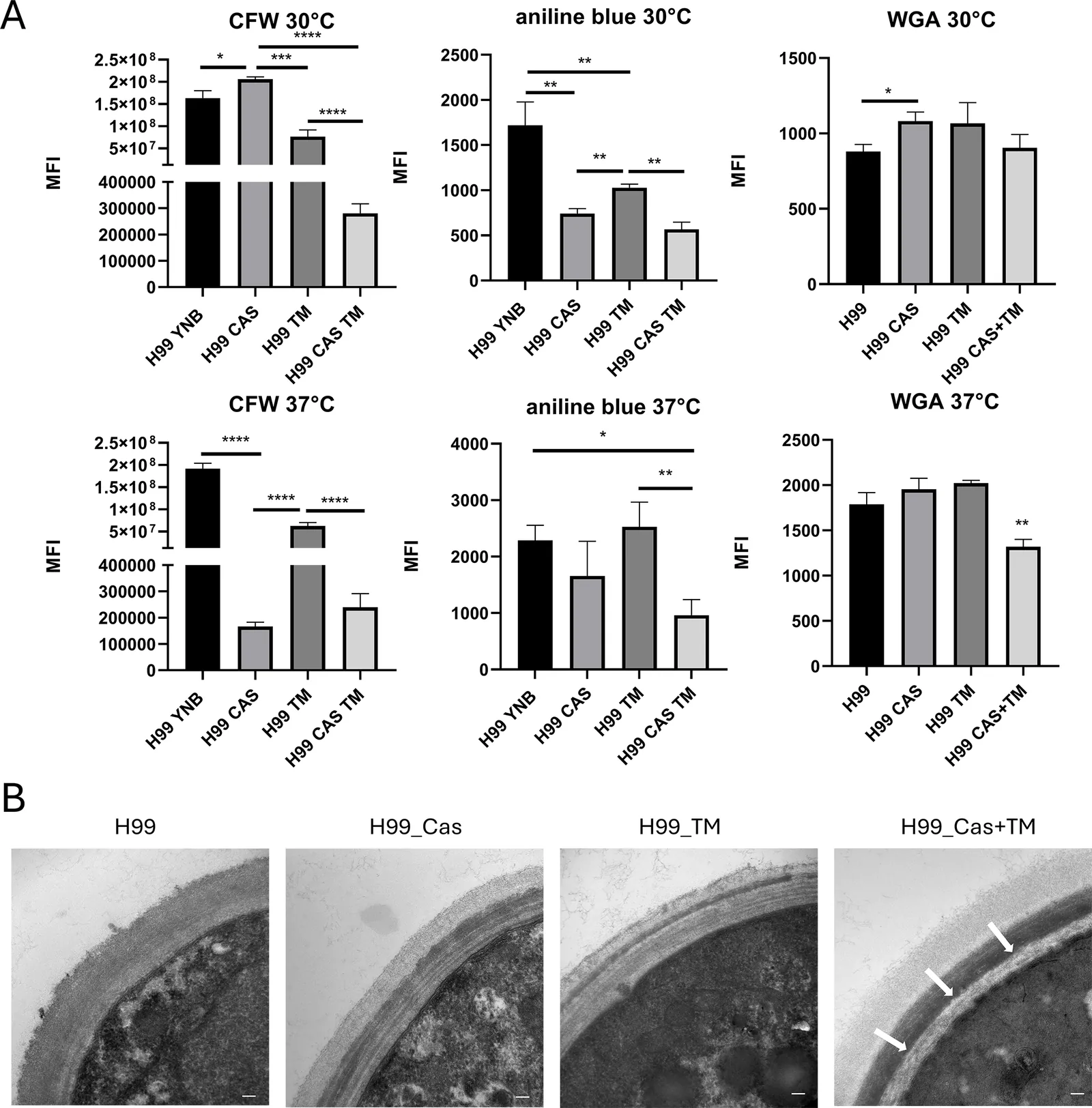
Cell wall defects induced by combined caspofungin and tunicamycin treatment. (**A**) levels of chitin (by calcofluor white (CFW) staining), β-glucan (aniline blue), and N-acetylglucosamine (detected with wheat germ agglutinin [WGA]) determined by flow cytometry with Pacific Blue (PB450, 405/450 nm), Pacific Blue (PB450, 405/450 nm), and fluorescein isothiocyanate (FITC, 488/530 nm), respectively. Cells were pre-grown in YPD and then transferred into minimal YNB medium with 10 µg/mL caspofungin (CAS), 125 ng/mL tunicamycin (TM), or with both drugs for 24 h. The assays were repeated three times with three biological replicates each. ΔMFI = change in mean fluorescence intensity. Error bars represent standard deviation. (**B**) Representative images (*n* = 10) of the cell walls obtained by transmission electron microscopy (TEM), scale bar = 100 nm. The arrows indicate regions that appear hollow in the inner cell wall. Cells were grown overnight in YPD, transferred to YNB with or without the indicated drug treatments (10 µg/mL CAS, 125 ng/mL TM) for 48 h at 30°C. Statistical significance was determined using one-way ANOVA with Tukey’s multiple comparisons in GraphPad Prism 8.0 (GraphPad Software) (*P < 0.05 **P < 0.01, ***P < 0.001, and ****P < 0.0001).

We next used transmission electron microscopy (TEM) to examine the cell wall structure of the WT strain with and without drug treatment. Upon treatment with both caspofungin and tunicamycin, the inner cell wall appeared damaged with hollow spaces, indicating structural disruption (Fig. 2B; Fig. S5). This region of the wall is reported to be primarily composed of β-1,3-glucan and chitin (23). Previous studies revealed that cell wall integrity and component distribution are influenced by environmental pH, with decreased chitin and β-glucan at pH 7 in YNB medium (44). To assess the influence of pH on the synergistic interaction between caspofungin and tunicamycin, we repeated the treatment experiments using YNB buffered to pH 7 with 50 mM 3-morpholinopropane-1-sulfonic acid (MOPS). The synergistic effect between caspofungin and tunicamycin specifically identified at 37°C was still observed in the buffered condition (Table S1).

We further investigated the effects of caspofungin and tunicamycin treatment on the endoplasmic reticulum (ER) and cell wall by examining the transcriptional response of key genes. Specifically, we analyzed transcript abundance after 2 h of treatment for genes involved in the unfolded protein response (UPR) pathway (*IRE1*, *HXL1_1*, *HXL1_2*), capsule formation (*CAP60*, *CAP64*), chitin synthesis (*CHS1–CHS8*), chitosan formation (*CDA1*, *CDA2*, *CDA3*), α-1,3-glucan synthesis (*AGS1*), β-1,3-glucan synthesis (*FKS1*), and β-1,6-glucan synthesis (*KRE6*, *SKN1*) (Fig. 3; Fig. S6). We found that transcripts for the UPR-related gene *HXL1-2* were significantly elevated, while those for capsule-related genes were reduced in response to the combination of caspofungin and tunicamycin, with less obvious changes for the treatment with each individual drug alone. We also found that caspofungin was sufficient to induce the upregulation of transcripts for several cell wall genes, including *HXL1_1*, *CHS2*, *CHS3*, *CHS4*, *CHS5*, *CHS7*, *CDA1*, and *CDA3*, especially at 30°C (Fig. 3A and B). These results are similar to the previously reported transcriptional response of cell wall genes to caspofungin (17), which might be compensatory responses to altered glycans levels. The transcript levels for the *CHS2*, *CHS5*, *CHS7*, and *CHS8*, were also elevated upon co-treatment with caspofungin and tunicamycin (Fig. 3B). In contrast, we also observed that the transcript levels for *CHS1*, *CDA2*, *CDA3, AGS1, KRE6,* and *SKN1* were reduced after co-treatment of caspofungin and tunicamycin, suggesting the addition of tunicamycin prevented this compensatory response to some extent. Similar trends were observed upon growth at 37°C (Fig. S6).

**Fig 3:**
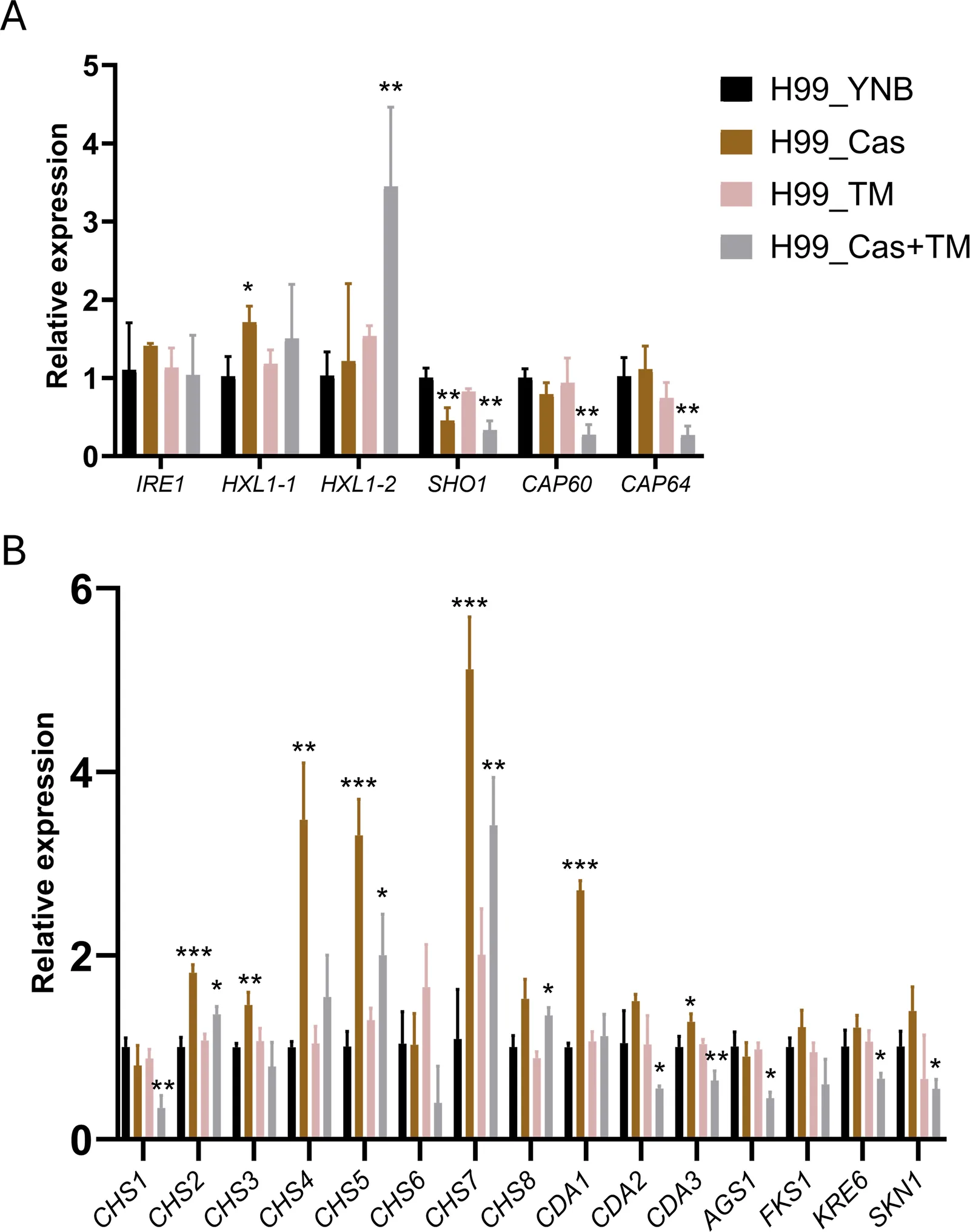
Cell wall gene expression is altered during caspofungin and tunicamycin treatment. qPCR analysis of the transcript levels for genes related to (**A**) the UPR and capsule or (**B**) cell wall synthesis upon growth in drug treatment conditions for 2 h at 30°C. The relative expression levels were compared between the different conditions for the WT strain H99 in YNB without or with 10 µg/mL caspofungin (CAS), 125 ng/mL tunicamycin (TM), or a combination of the two, using the housekeeping gene *GAPDH* as a control. The assays were performed twice with three biological replicates each. The mean and standard deviation of the measurements are shown. Statistical significance was determined by performing one-way ANOVA and Tukey’s multiple comparisons in GraphPad Prism 8.0 (GraphPad Software) (*P < 0.05 **P < 0.01 and ***P < 0.001; the comparisons were all to the WT strain in YNB alone).

Membrane integrity is involved in the resistance of *C. neoformans* to caspofungin, and SDS was found to have synergistic effects with the drug (16). Cells treated with caspofungin and tunicamycin in combination for 48 h exhibited subtly increased sensitivity on agar medium containing both drugs (Fig. 4A). The cells also showed mildly increased sensitivity to membrane and cell wall stress (i.e., growth on 0.005% SDS or caffeine) compared with untreated controls or single drug treatments (Fig. 4A). We also observed that the fungal cells appeared smaller following treatment with caspofungin and tunicamycin in YNB medium (Fig. 4B and C). To further investigate membrane integrity, cells previously exposed to caspofungin or tunicamycin (alone or in combination) were incubated for an additional 3 h at 30°C in YNB with or without 0.005% SDS. Propidium iodide (PI) staining was used to assess cell viability and membrane permeability (Fig. 4D). Cells treated with the drug combination showed increased PI uptake, indicating compromised membrane integrity. These results suggest that combined exposure to caspofungin and tunicamycin damages both the cell wall (Fig. 2) and the plasma membrane. In this regard, the results are also consistent with reduced cell viability. Interestingly, the calcium channel inhibitor verapamil also has synergy with caspofungin in *C. neoformans* and influences cell wall and membrane integrity (15, 27). We therefore determined whether the combination of tunicamycin and caspofungin influenced calcium levels and, indeed, found elevated intracellular calcium after 48 h of treatment (Fig. 4E). This result suggests that the combined drug treatment may impact calcium signaling and could synergize with verapamil.

**Fig 4:**
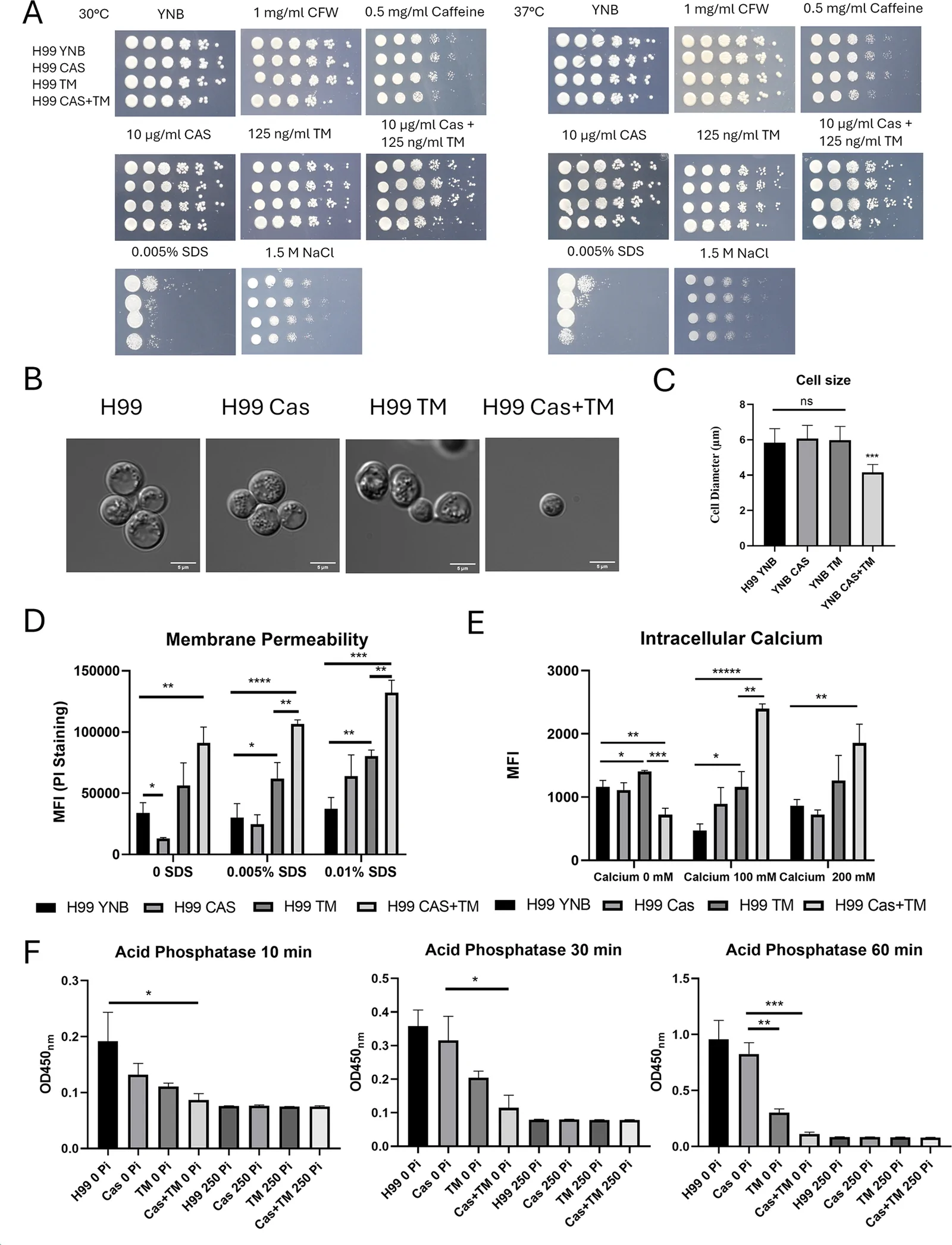
Disruption of membrane integrity and intracellular calcium levels by tunicamycin and caspofungin (**A**) Growth assays to test drug resistance using tunicamycin (TM), caspofungin (CAS), SDS, NaCl, calcofluor white (CFW), and caffeine. Plates were incubated at 30°C or 37°C for 3 days and then photographed. The assays were repeated three times. (**B**) Differential interference contrast microscopy (DIC) of WT strain (H99) grown at 30°C in YNB with or without drug (10 µg/mL CAS, 125 ng/mL TM) for 48 h (scale bar = 5 µm). (**C**) Measurement of cell size from panel **B** (*n* = 60). Two independent experiments were conducted, and the mean and standard deviation of measurements are shown. (**D**) Viability and membrane permeability were determined with propidium iodide (PI) staining, followed by flow cytometry with FITC, 488/530 nm. Cells were cultured in YNB medium for 48 h with either single or combined treatment of CAS and TM. Then transferred into fresh YNB with or without 0.005% SDS for 3 h at 30°C. OD_600_ = 1 cells were harvested and used for staining. (**E**) Levels of intracellular calcium were determined with calcium indicator Cal Green 1 AM (Calcium Green-1 AM) staining and detected by FITC, 488/530 nm channel using flow cytometry. Cells were cultured in YNB medium for 48 h with either single or combined treatment of CAS and TM, then transferred into fresh YNB with or without 100 mM or 200 mM CaCl_2_ for 3 h at 30°C. OD_600_ = 1 cells were harvested and used for staining. (**F**) Assays for extracellular acid phosphatase activity at the indicated times for cells grown in different levels of phosphate. Cells were grown overnight in YPD, and transferred into four treatment conditions: fresh YNB, YNB with 10 µg/mL caspofungin, YNB with 125 ng/mL tunicamycin, YNB containing both caspofungin and tunicamycin for 48 h at 30°C. Cells were then resuspended in MM-250 mM KH_2_PO_4_ or MM-0 Pi medium, incubated at 30°C for 3 h. After incubation, supernatants (50 µL) of each sample were collected and used to measure acid phosphatase activity using the Acid Phosphatase Assay Kit from Sigma Aldrich (MAK446). Statistical significance was determined by performing one-way ANOVA and Tukey’s multiple comparisons in GraphPad Prism 8.0 (GraphPad Software) (*P < 0.05 **P < 0.01, ***P < 0.001 and ****P < 0.0001).

The link between membrane integrity and caspofungin resistance has also been studied with the lipid flippase Apt1 and the regulatory subunit Cdc50 (located in the ER), which maintain the asymmetry of plasma membrane phospholipids. For example, defects in these functions influence susceptibility to echinocandins, and the natural product ButA inhibits Apt1 and affects caspofungin resistance (24, 34, 38). This result is consistent with the importance of flippase activity and membrane composition (34). In this context, we found that mutants lacking Cdc50, Apt1, or Apt4 (another P-type ATPase) had increased sensitivity to combined treatment with caspofungin and tunicamycin (Fig. S7). Taken together, our results suggest that caspofungin resistance and membrane integrity are closely associated with the proper localization of Fks1 at the plasma membrane.

### Caspofungin and tunicamycin together alter/influence protein trafficking to the cell surface

To further investigate the combined impact of the drugs, we measured acid phosphatase activity to assess whether treatment interfered with delivery of proteins to the cell surface (Fig. 4F). We found that activity was lower upon treatment with each drug individually and was further reduced by the combination. The treatments were performed with cells grown in low phosphate medium to induce expression of the enzyme and, as a control, growth in high phosphate substantially reduced activity to levels similar to caspofungin plus tunicamycin treatment. We also examined the localization of the caspofungin target 1,3-β-glucan synthase. A previous study with Fks1-GFP revealed heterogeneity for the signal with punctate structures distributed on the cell membrane, enriched at the plasma membrane, and partially present in the perinuclear endoplasmic reticulum (ER) (17, 45). This localization is not altered during caspofungin treatment (17). Consistent with these findings, our results confirm that after 24 h of culture in YNB medium, Fks1-GFP accumulated on the plasma membrane and is partially associated with the ER, which may correspond to vesicular compartments involved in secretion and trafficking. This distribution remained unchanged after caspofungin treatment (Fig. 5; Fig. S8). However, tunicamycin treatment leads to a redistribution of Fks1-GFP with reduced ER-associated signal and a more pronounced punctate distribution within the cell. Combined treatment with tunicamycin and caspofungin further enhanced the punctate distribution phenotype. To compare the localization, we used Sec61-GFP, which is known as ER membrane protein. Tunicamycin alone or combined with caspofungin did not alter the localization of Sec61. We also found the fluorescence intensity of Fks1-GFP was appreciably lower than that of the endoplasmic reticulum marker Sec61 (Fig. 5; Fig. S8).

**Fig 5:**
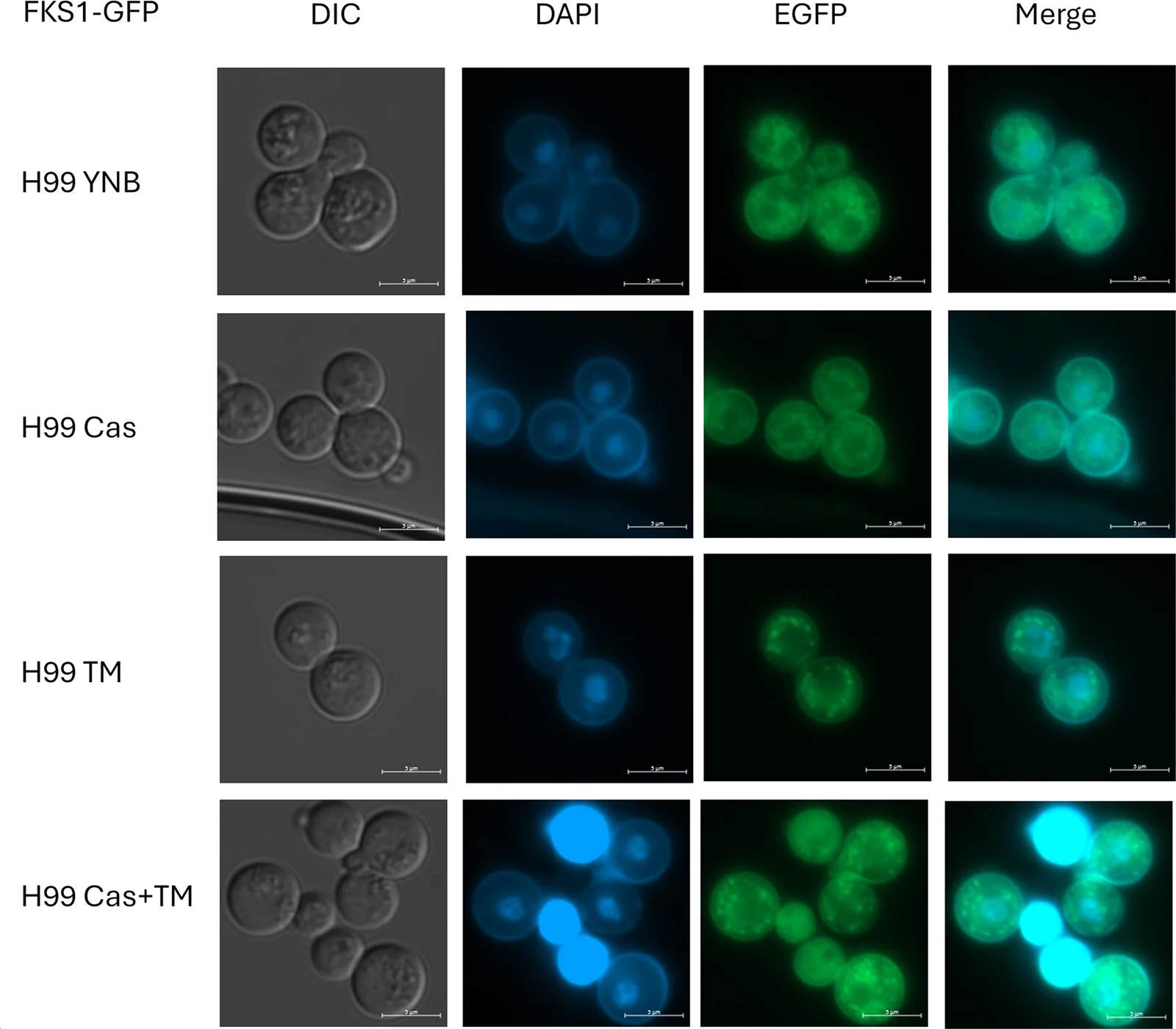
Fks1-GFP localization is altered during combined treatment with both caspofungin and tunicamycin. FKS1-GFP cells were grown overnight in YPD and then transferred into four 3 mL treatment conditions: fresh YNB, YNB with 10 µg/mL caspofungin (CAS), YNB with 125 ng/mL tunicamycin (TM), YNB containing both CAS and TM for 24 h at 30°C. Cells at OD_600_ = 1 were collected and fixed with 2% paraformaldehyde (PFA) at RT for 20 min, then stained with 5 µm/mL DAPI (4′,6-diamidino-2-phenylindole) for nuclei at RT in the dark for 30 min. Cells were imaged using a Zeiss Plan-Apochromat 100×/1.46 oil lens on a Zeiss Axioplan 2 microscope.

### Cells treated with caspofungin and tunicamycin have altered uptake and proliferation in phagocytes

Caspofungin disrupts the synthesis of β-1,3-glucan, triggering compensatory cell wall remodeling, which alters the recognition and uptake of fungi by macrophages (28, 29). We therefore tested how the combination of tunicamycin and caspofungin affects fungal uptake and proliferation in macrophages (Fig. 6). Infection experiments with the J774A.1 macrophage-like cell line were initiated with cryptococcal cells pretreated with tunicamycin or caspofungin alone or the two drugs in combination, during growth in YNB media for 48 h. The uptake of yeast cells was quantified by washing off extracellular yeast cells for half of the macrophages at 2 h post-infection and counting CFUs. Further incubation of the remaining macrophages allowed intracellular proliferation to be assessed by counting CFUs at 24 h post-infection. The capsule-binding monoclonal antibody 18B7 that facilitates opsonization enhanced uptake relative to no antibody treatment, and the higher levels of uptake generally correlated with greater intracellular proliferation for all treatments (Fig. 6). Exceptions were noted for the cells treated with tunicamycin alone or with both drugs where 18B7 did not markedly enhance uptake at 2 h compared with the untreated cells, perhaps indicating an influence of cell surface changes on antibody binding. Cells treated with tunicamycin alone showed reduced uptake in the presence of the antibody and comparable intracellular proliferation to treatment with caspofungin. Treating the cells with caspofungin alone did not have a substantial influence on uptake or proliferation with or without 18B7 treatment. Relative to untreated cells, or cells treated with either drug alone, lower levels of uptake and proliferation were observed upon treatment with both caspofungin and tunicamycin. However, substantial proliferation was observed given the lower level of uptake for the cells treated with both drugs (Fig. 6). Together, these results reveal an impact of tunicamycin and the combined treatment with both drugs on uptake and proliferation during interactions with phagocytes. This impact may in part reflect influences of the drugs on the cell surface and recognition by anti-capsule antibody and phagocytes.

**Fig 6:**
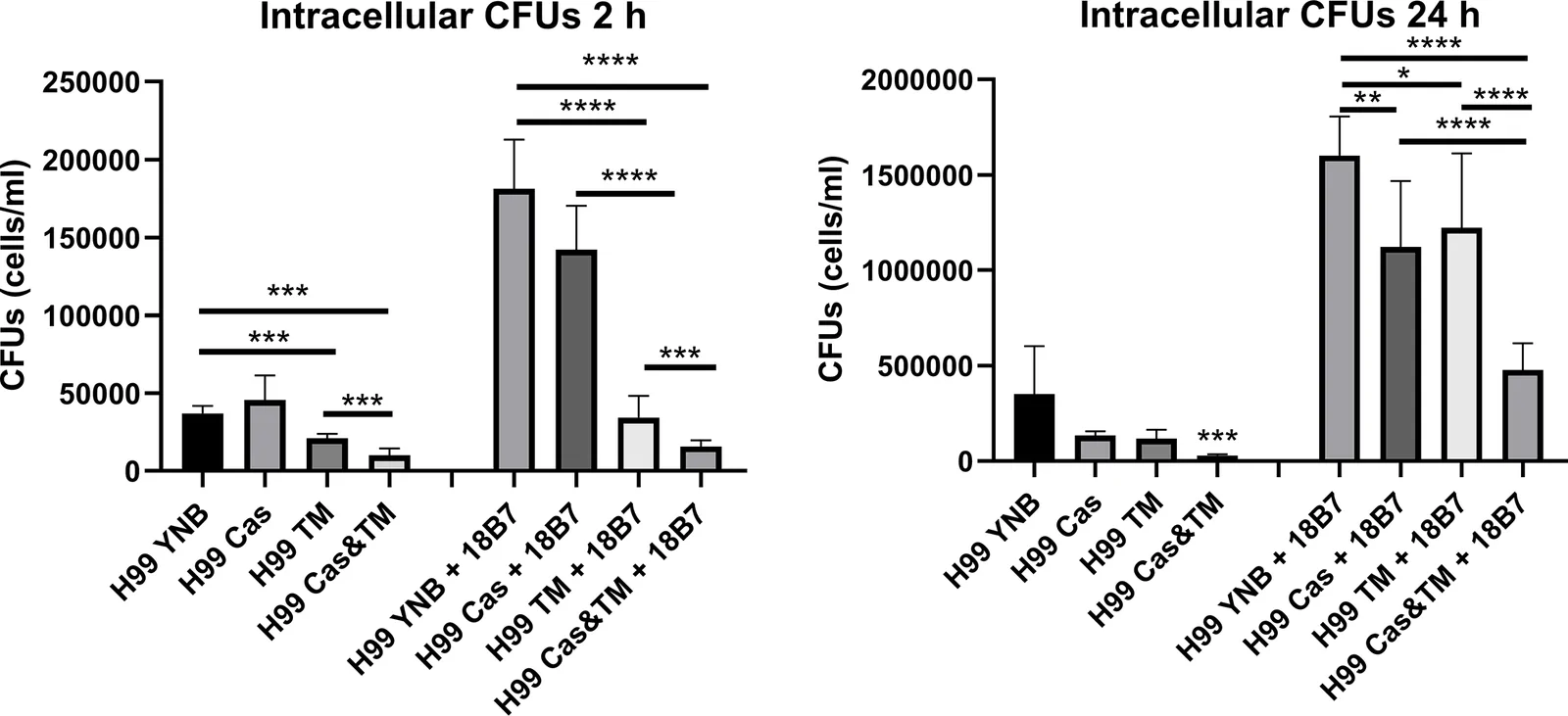
Cells treated with tunicamycin and caspofungin show impaired interactions with phagocytic cells. Intracellular CFUs of WT cryptococcus cells treated as indicated were collected at 2 h post-infection (phagocytosis) and 24 h post-infection (proliferation). After 2-h incubation, external yeast cells were washed off with sterile PBS (4 or 5 washes), and half of the macrophages were lysed with sterile distilled water to collect intracellular fungal cells. After 24 h of incubation, sterile, ice‐cold distilled H_2_O was applied to another half to lyse the macrophages. Fungal growth was measured by plating cells on YPD and determining CFUs. Statistical significance was determined by performing one-way ANOVA and Tukey’s multiple comparisons in GraphPad Prism 8.0 (GraphPad Software) (*P < 0.05, **P < 0.01, ***P < 0.001, and ****, P < 0.0001).

### Evaluation of additional ER inhibitors for potential synergy with caspofungin

To further investigate the synergistic effects between caspofungin and endoplasmic reticulum (ER) stress pathways, we screened the TargetMol Endoplasmic Reticulum (ER) Stress Compound Library (https://www.targetmol.com/compound-library/inhibitor_library). We combined these ER stress-related compounds with caspofungin at concentrations of 20 µg/mL (Fig. 7A) and 10 µg/mL (Fig. 7B) to assess relative fungal growth up to 72 h (Table S2). Several compounds showed inhibitory effects on *C. neoformans* (Fig. 7), highlighting their potential as promising candidates for further investigation in antifungal drug development. We selected RA190, honokiol, HSP990, pyrazolanthrone, timosaponin AIII, CREB inhibitor, and polyphyllin I for FIC analysis to identify potential synergy. The MIC_50_ of each compound was determined in combination with caspofungin in a range from 0 to 20 µg/mL and the ER stress inducers in a range from 0.625 to 20 µM (Table 3). Depending on the criteria for synergy described above for tunicamycin, our results suggested potential synergy for RA190, honokiol, HSP990, timosaponin AIII, and polyphyllin I with caspofungin at 30°C, while only HSP990 and timosaponin AIII had potential synergistic effects at 37°C (Table 3). Similar results were obtained when MIC_90_ values were examined (Table 4). In addition, we examined the influence on capsule formation and cell wall composition with 2.5 µM of the compounds and 10 µg/mL caspofungin (Fig. 8). RA190, honokiol, HSP990, and pyrazolanthrone alone inhibited capsule formation, and inhibition was enhanced when combined with caspofungin.

**Fig 7:**
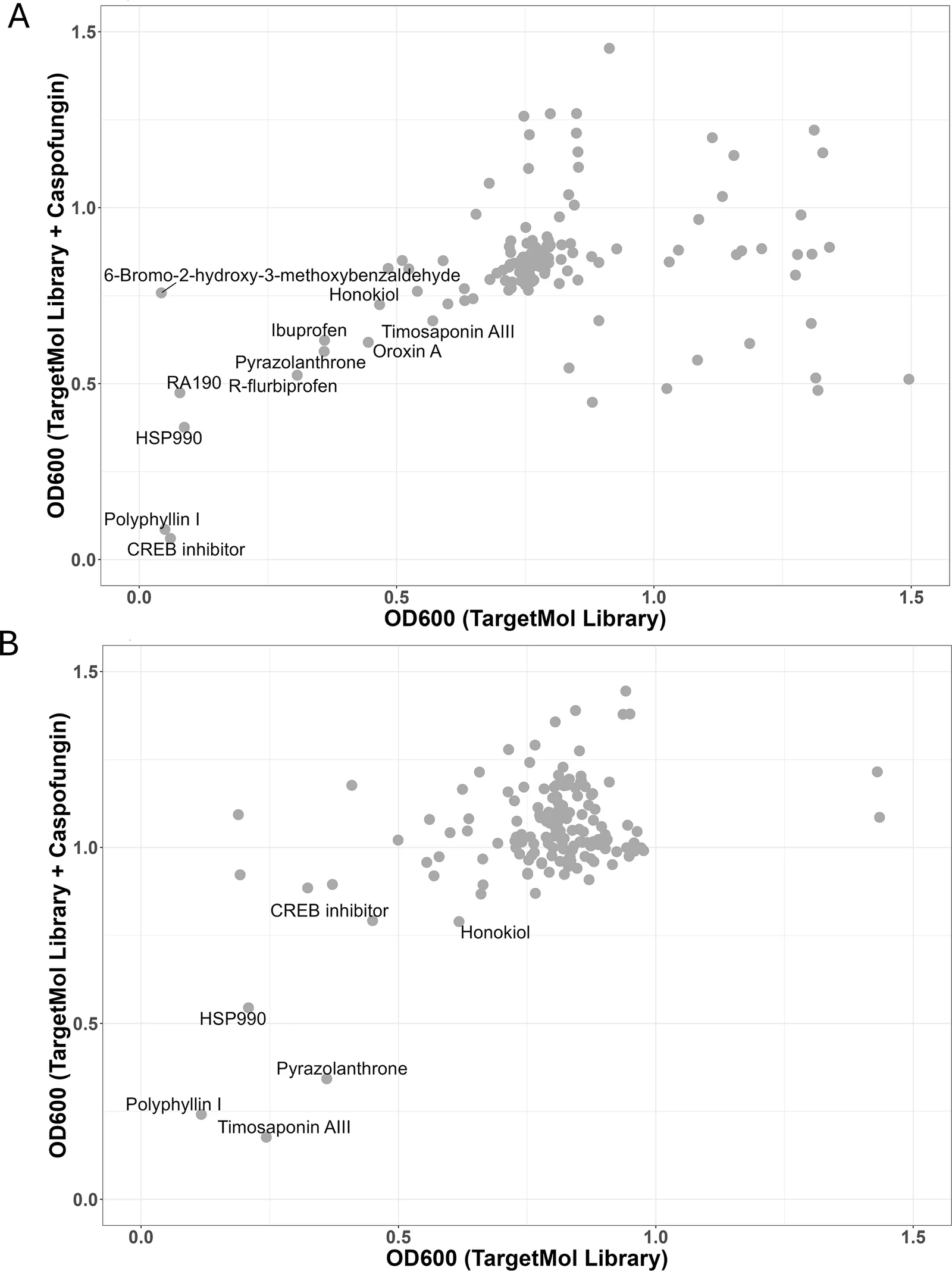
A screen of an ER inhibitor library identifies additional molecules that enhance sensitivity to caspofungin. Cells of the WT H99 strain were grown overnight in YPD at 30°C, washed, and transferred into YNB medium in 96-well plates for growth assays. TargetMol endoplasmic reticulum (ER) Stress Compound Library (https://www.targetmol.com/compound-library/inhibitor_library) was tested with 10 µM. Caspofungin was tested in a concentration of 10 (**A**) and 20 µg/mL (**B**). OD_600_ was measured every 24 h, and 72-h results are shown. Each condition was tested in three independent experiments with three biological replicates. The OD_600_ measurements of the replicates can be found in Table S2.

**Fig 8:**
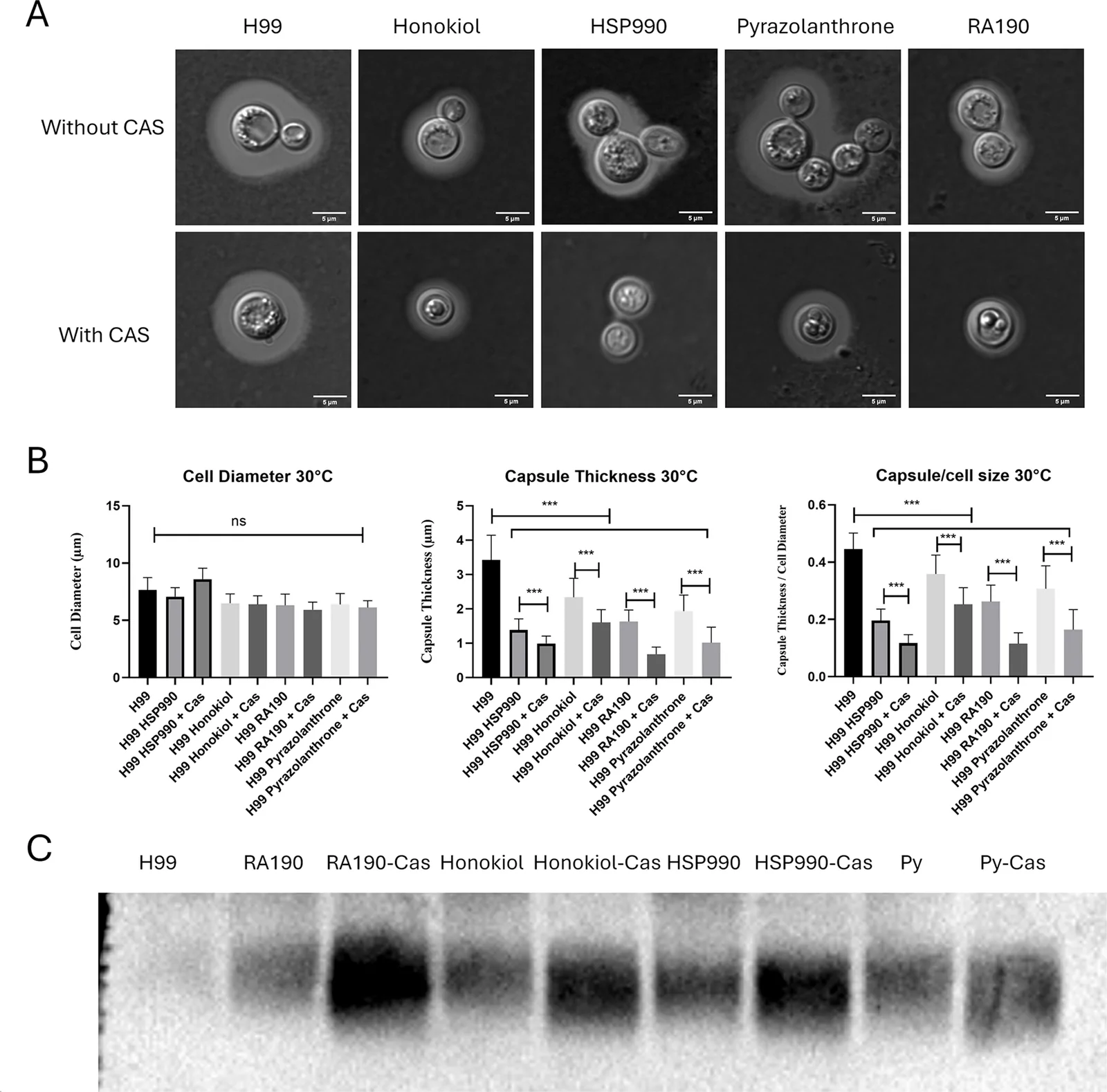
Treatment with candidate ER inhibitors and caspofungin provoke capsule defects. (**A**) Differential interference contrast microscopy (DIC) of capsule stained with India ink for the WT strain (H99) grown at 30°C in LIM with different treatment (RA190, honokiol, HSP990, and pyrazolanthrone (all at 5 µM) and caspofungin (CAS at 10 µg/mL) for 48 h (scale bar = 5 µm). Note that the representative H99 cell with CAS treatment was also part of the experiment shown in Fig. 1A. (**B**) Measurement of cell size, capsule thickness, and ratio of capsule/cell size from panel **A** (*n* = 60). Three independent experiments were conducted, and the mean and standard deviation of measurement are shown. (**C**) Immunoblot of shed capsule polysaccharide in culture supernatants after 48 h of growth in LIM. Three biological replicates are shown, and the assays were repeated twice. Statistical significance was determined by performing one-way ANOVA and Tukey’s multiple comparisons in GraphPad Prism 8.0 (GraphPad Software) (ns, not significant; ***P < 0.001).

**TABLE 3:** Minimal inhibitory concentration (MIC_50_) and fractional inhibitory concentration (FIC) index (FIC) assays (caspofungin with other ER inhibitors) using strain H99^*c*^

Temp	MIC_50_				FIC	Relationship(s)
	Alone		Combined			
30°C	RA190, 25 μM	Caspofungin, 20 μg/mL	RA190, 5 μM	Caspofungin, 10 μg/mL	0.7	Synergy,^*a*^ indifference^*b*^
	Honokiol, 40 μM	Caspofungin, 20 μg/mL	Honokiol, 15 μM	Caspofungin, 10 μg/mL	0.875	Synergy,^*a*^ indifference^*b*^
	HSP990, 5 μM	Caspofungin, 20 μg/mL	HSP990, 1.25 μM	Caspofungin, 10 μg/mL	0.75	Synergy,^*a*^ indifference^*b*^
	Pyrazolanthrone, 10 μM	Caspofungin, 20 μg/mL	Pyrazolanthrone, 5 μM	Caspofungin, 10 μg/mL	1	Indifference^*b*^
	Timosaponin AIII, 10 μM	Caspofungin, 20 μg/mL	Timosaponin AIII, 2.5 μM	Caspofungin, 10 μg/mL	0.75	Synergy,^*a*^ indifference^*b*^
	CREB inhibitor, 20 μM	Caspofungin, 20 μg/mL	CREB inhibitor, 10 μM	Caspofungin, 10 μg/mL	1	Indifference^*b*^
	Polyphyllin I, 5 μM	Caspofungin, 20 μg/mL	Polyphyllin I, 2.5 μM	Caspofungin, 5 μg/mL	0.75	Synergy,^*a*^ indifference^*b*^
37°C	RA190, 10 μM	Caspofungin, 15 μg/mL	RA190, 5 μM	Caspofungin, 10 μg/mL	1.17	Indifference^*b*^
	Honokiol, 15 μM	Caspofungin, 15 μg/mL	Honokiol, 5 μM	Caspofungin, 10 μg/mL	1	Indifference^*b*^
	HSP990, 5 μM	Caspofungin, 15 μg/mL	HSP990, 1.25 μM^*c*^	Caspofungin, 10 μg/mL	0.85	Synergy,^*a*^ indifference^*b*^
	HSP990, 5 μM	Caspofungin, 15 μg/mL	HSP990, 2.5 μM^*c*^	Caspofungin, 5 μg/mL	0.83	Synergy,^*a*^ indifference^*b*^
	Pyrazolanthrone, 10 μM	Caspofungin, 15 μg/mL	Pyrazolanthrone, 5 μM	Caspofungin, 10 μg/mL	1.17	Indifference^*b*^
	Timosaponin AIII, 10 μM	Caspofungin, 15 μg/mL	Timosaponin AIII, 2.5 μM	Caspofungin, 10 μg/mL	0.92	Synergy,^*a*^ indifference^*b*^
	CREB inhibitor, 20 μM	Caspofungin, 15 μg/mL	CREB inhibitor, 10 μM	Caspofungin, 10 μg/mL	1.17	Indifference^*b*^
	Polyphyllin I, 5 μM	Caspofungin, 15 μg/mL	Polyphyllin I, 2.5 μM	Caspofungin, 10 μg/mL	1.17	Indifference^*b*^

^*a*^ FICI <1.0 as synergy, =1.0 as additivity, and 1.0–2.0 as autonomous (15, 17, 39).

^*b*^ FICI ≤0.5 as synergy, >0.5–4.0 as indifference, and >4.0 as antagonistic (14, 16, 25, 27, 34).

^*c*^ There are two combinations of HSP990 and caspofungin which showed similar FIC values, and both are shown.

**TABLE 4:** Minimal inhibitory concentration (MIC_90_) and fractional inhibitory concentration (FIC) index assays (caspofungin with other ER inhibitors) using strain H99

Temp	MIC_90_				FIC	Relationship(s)
	Alone		Combined			
30°C	RA190, 25 μM	Caspofungin, 25 μg/mL	RA190, 5 μM	Caspofungin, 10 μg/mL	0.6	Synergy,^*a*^ indifference^*b*^
	Honokiol, 50 μM	Caspofungin, 25 μg/mL	Honokiol, 25 μM	Caspofungin, 20 μg/mL	1.3	Indifference^*b*^
	HSP990, > 20 µM	Caspofungin, 25 μg/mL	HSP990, 5 μM	Caspofungin, 10 μg/mL	0.6	Synergy,^*a*^ indifference^*b*^
	Pyrazolanthrone, 10 μM	Caspofungin, 25 μg/mL	Pyrazolanthrone, 5 μM	Caspofungin, 10 μg/mL	0.9	Synergy,^*a*^ indifference^*b*^
	Timosaponin AIII, >20 µM	Caspofungin, 25 μg/mL	Timosaponin AIII, 10 μM	Caspofungin, 10 μg/mL	0.9	Synergy,^*a*^ indifference^*b*^
	CREB inhibitor, >20 µM	Caspofungin, 25 μg/mL	CREB inhibitor, 20 μM	Caspofungin, 10 μg/mL	1.4	Indifference^*b*^
	Polyphyllin I, 10 μM	Caspofungin, 5 μg/mL	Polyphyllin I, 5 μM	Caspofungin, 10 μg/mL	0.9	Synergy,^*a*^ indifference^*b*^
37°C	RA190, 10 μM	Caspofungin, 20 μg/mL	RA190, 5 μM	Caspofungin, 10 μg/mL	1	Indifference^*b*^
	Honokiol, 30 μM	Caspofungin, 20 μg/mL	Honokiol, 15 μM	Caspofungin, 10 μg/mL	1	Indifference^*b*^
	HSP990, 5 μM	Caspofungin, 20 μg/mL	HSP990, 1.25 μM	Caspofungin, 10 μg/mL	0.75	Synergy,^*a*^ indifference^*b*^
	Pyrazolanthrone, 10 μM	Caspofungin, 20 μg/mL	Pyrazolanthrone, 5 μM	Caspofungin, 10 μg/mL	1	Indifference^*b*^
	Timosaponin AIII, >20 µM	Caspofungin, 20 μg/mL	Timosaponin AIII, 10 μM	Caspofungin, 20 μg/mL	1.5	Indifference^*b*^
	CREB inhibitor, 20 μM	Caspofungin, 20 μg/mL	CREB inhibitor, 20 μM	Caspofungin, 5 μg/mL	1.25	Indifference^*b*^
	Polyphyllin I, 10 μM	Caspofungin, 20 μg/mL	Polyphyllin I, 5 μM	Caspofungin, 5 μg/mL	0.75	Synergy,^*a*^ indifference^*b*^

^*a*^ FICI <1.0 as synergy, =1.0 as additivity, and 1.0–2.0 as autonomous (15, 17, 39).

^*b*^ FICI ≤0.5 as synergy, >0.5–4.0 as indifference, and >4.0 as antagonistic (14, 16, 25, 27, 34).

In a parallel genetic approach, we also examined connections between caspofungin and ER function using previously characterized mutants with ER defects. Specifically, Dnj1 is a J-domain containing co-chaperone involved in maintaining ER homeostasis (46), and Cne1 is a calcium-binding ER chaperone protein that ensures proper folding of glycoproteins (47). Both proteins contribute to the response to ER stress. We found that mutants lacking Dnj1 were similar to the WT in sensitivity to combined treatment with caspofungin and tunicamycin (Fig. S9). In contrast, the *cne1*Δ mutant showed reduced growth when treated with 10 μg/mL caspofungin, and further impaired growth when treated with 5 μg/mL caspofungin and 125 ng/mL tunicamycin, exhibiting higher sensitivity than the WT strain or the *dnj1*Δ mutant (Fig. S9). Thus, Dnj1 and Cne1 play distinct roles, and the relationship between Cne1 and caspofungin may point to a specific impact of protein folding.

## DISCUSSION

The mechanisms by which *C. neoformans* resists echinocandin drugs have been investigated in several studies with the emerging theme that resistance is influenced by membrane integrity and membrane-associated processes, such as drug efflux (16–18, 24, 48–50). The influence of membrane integrity is apparent from the observation that SDS treatment has a synergistic effect with caspofungin (16). Furthermore, the lipid flippase Apt1 and the regulatory subunit Cdc50 (located in the ER) maintain the asymmetry of plasma membrane phospholipids and influence susceptibility to echinocandins (24, 34, 38). The importance of flippase activity and membrane composition is highlighted by the discovery that the natural product ButA inhibits Apt1 and ablates caspofungin resistance (34). Additionally, the efflux inhibitor clorgiline strongly synergizes with caspofungin (17). Consistent with these findings, we identified synergy between caspofungin and tunicamycin leading to lower MICs, increased sensitivity to SDS, and enhanced cell membrane permeability. Tunicamycin itself has been identified as a potential antifungal agent, and interactions with other antifungal drugs have been reported. For example, exposure to tunicamycin affects fluconazole tolerance in *Nakaseomyces glabratus* (formerly *C. glabrata*), and tunicamycin and amphotericin B act synergistically to inhibit functions involved in mannoprotein or ergosterol biosynthesis in *Candida* species (51, 52). Furthermore, aneuploidy promotes adaptation to tunicamycin-induced ER stress and confers cross-resistance to caspofungin in multiple yeast species including *Saccharomyces cerevisiae, C. albicans* and *C. neoformans* (52–55).

The underlying mechanism that connects caspofungin resistance to membrane integrity may reflect the importance of the interaction of the target enzyme Fks1 with the plasma membrane. Fks1 is a transmembrane heteromeric glycosyltransferase using UDP-glucose as a substrate (6, 56). Elegant structural studies indicate that the enzyme induces thinning of the outer leaflet of the membrane and that caspofungin acts at the membrane-protein interface (50, 57, 58). There is also evidence that caspofungin interacts with cardiolipins in the mitochondrial membrane (59). Caspofungin induces dynamic changes in the localization of an Fks1-GFP fusion in *A. fumigatus*, with shifts between growing hyphal tips to vacuoles (60). Localization experiments in *C. neoformans* found Fks1-GFP enriched in the plasma membrane in most of the cells, and partially present in the perinuclear ER (17, 45). This localization is not altered during caspofungin treatment (17). Our localization experiments align with these previous results, although we noticed that tunicamycin skewed localization toward punctate locations in the cytoplasm, and combined drug treatment also revealed this pattern. This mislocalization reinforces the idea that the drugs impair ER-mediated trafficking, leading to changes in cell wall and membrane integrity; our observed decrease of acid phosphatase secretion after combined treatment is consistent with this conclusion.

In *C. neoformans,* the Fks1 β-1,3-glucan synthase is sensitive to low concentrations of caspofungin *in vitro* (8). Additionally, a reduction in the abundance of Fks1 at the cell wall is known to increase sensitivity to echinocandin drugs (61). It is possible the resistance may result in part from the capsule and cell wall impeding access of the caspofungin to Fks1 (7, 22, 23). We found that combined treatment reduced capsule thickness and increased capsule shedding, indicating impaired capsule attachment. Previous studies have shown that acapsular mutants exhibit caspofungin resistance comparable to WT cells (24), and our results showed that acapsular mutants exhibited similar changes in cell wall composition, compared with WT, suggesting that the capsule is not the cause of intrinsic resistance to caspofungin in *C. neoformans*. The increase in capsule shedding is likely due to altered cell wall composition due to defects in delivering cell wall biosynthetic enzymes. In particular, capsule attachment is attributed to α−1,3-glucans (62). Our investigation of the effects of combined treatment with caspofungin and tunicamycin identified changes in cell wall structure. Previous studies have shown that caspofungin alone reduces β-1,3-glucan and β-1,6-glucan levels but results in increased chitin and chitosan levels in *C. neoformans* and *Candida* species (17, 20, 41, 42). Consistent with these findings, we observed similar changes after treatment with caspofungin alone. However, when caspofungin was combined with tunicamycin, both chitin and β-glucan levels decreased, indicating significant changes in cell wall composition, particularly within the inner cell wall layer.

Loss of the protein kinase C or calcineurin pathways also affect tolerance to both tunicamycin and caspofungin, suggesting that ER stress and echinocandin resistance are regulated by overlapping stress response networks (54, 63, 64). Caspofungin treatment has been shown to increase intracellular calcium levels in human respiratory epithelial cells by inducing release from internal stores (65). The relationship between calcium homeostasis and caspofungin response has also been reported in fungi, where activation of the calcium-calcineurin pathway increases chitin content and reduces susceptibility to caspofungin in *C. albicans* and *A. fumigatus* to caspofungin (63, 66–69). In *C. neoformans*, intracellular Ca²^+^ levels were significantly increased in the *cdc50*Δ mutant compared with the WT, supporting a role for calcium dysregulation in caspofungin susceptibility (70, 71). Calcineurin inhibitors FK506 and calcium channel inhibitor verapamil were found to have a synergistic effect with caspofungin in *C. neoformans* (15, 27). The combination of verapamil and caspofungin altered cell wall integrity, membrane permeability, ROS accumulation, and intracellular calcium concentration (27). Consistent with these findings, our results showed that intracellular Ca²^+^ concentrations were increased in cells upon combined treatment, indicating a disruption of calcium homeostasis, which may contribute to the observed synergistic antifungal activity.

The fungal cell wall is a dynamic structure composed primarily of β-1,3-D-glucan, β-1,4-D-glucan, β-1,6-D-glucan, α-glucan, chitin, mannan, and glycoproteins (7, 72). The β-1,3-D-glucan is the most abundant in many fungi, including *C. albicans* and *A. fumigatus* (7, 73, 74). In contrast, *C. neoformans* has a relatively low β-1,3-D-glucan content, with β-1,6-glucan being the main component of its cell wall (7, 18, 19). This compositional difference may contribute to the intrinsic resistance of *C. neoformans* to echinocandin drugs, as the lower β-1,3-glucan content reduces the accessibility of the drug to its target (7, 18, 19). The “cell wall paradox” refers to the fact that fungi compensate for β-glucan inhibition by upregulating chitin synthesis in response to environmental stress, nutrient limitations, or genetic perturbations (75–77). This paradox caused by caspofungin has been studied in *Candida* and *Aspergillus* species (42, 78–81). Our results with caspofungin alone were consistent with this finding. However, combined treatment with caspofungin and tunicamycin appeared to inhibit the compensatory increase in chitin, leading to increased cellular stress and severe cell wall damage. In general, loss of glycosylation likely impairs the delivery and function of cell wall biosynthetic enzymes leading to cell wall changes that may allow more access of caspofungin to Fks1 (leading to additional changes in cell wall composition).

Caspofungin-induced cell wall remodeling is known to affect host-pathogen interactions by reducing β-glucan content, unmasking cell wall β-glucan, and increasing chitin synthesis and exposure, which is usually associated with increased immune recognition (28, 76). Our results suggest that combination treatment, but not caspofungin alone, reduces macrophage phagocytosis and intracellular proliferation of *C. neoformans* with antibody opsonization, which could be due to reduced capsule after combined treatment. We also noticed a similar reduction of phagocytosis without antibody opsonization, which we think might be related to the reduction of chitin and β-1,3-glucan levels after combination treatment. Alternatively, the reduced phagocytosis may be due to the decrease in fungal viability, as a significant decrease in total CFU was observed in the combination treatment group.

The host response to cell wall remodeling varies among different species. In *Candida* species, caspofungin-induced cells show increased exposure of chitin and β-glucan, which is associated with decreased macrophage uptake and reduction in immune cell cytokine production (28, 43, 82). In *A. fumigatus*, echinocandin treatment of hyphae showed enhanced immune recognition and cytokine signaling by macrophages (29). Changes in cell wall composition were found to affect host responses in *C. neoformans*. Cells grown in unbuffered YNB medium, where the pH drops during incubation, exhibited higher levels of chitin and β-1,3-glucan, and were less virulent; mice infected with these cells survived longer compared with those infected with cells grown in buffered YNB (pH 7), which had lower levels of chitin and β-1,3-glucan (44). Titan cell production in *C. neoformans* showed increased capsule and reduced β-glucan both *in vivo* and *in vitro,* which is another reason to change the response to immune attack by masking cell wall β-glucan and chitin during infection in host (83). These findings further support the idea that cell wall composition influences host immune interactions (44).

Our screen of a library of ER inhibitors revealed additional ER stress inducers, with potential use for antifungal development. In particular, RA190, honokiol, HSP990, pyrazolanthrone, timosaponin AIII, CREB inhibitor, and polyphyllin I showed activity in ablating caspofungin resistance. These agents have a variety of activities. For example, honokiol inhibits the activation of Akt and induces autophagy by activating the phosphorylation of ERK1/ERK2 signaling and has antifungal activity on human pathogenic fungi and plant fungal diseases (84–87). Honokiol and fluconazole act synergistically against *C. albicans* by disrupting the cell membrane (88, 89). HSP990 is an HSP90α/β inhibitor with antitumor activities and is found to have a synergistic effect with fluconazole against *C. albicans* and *C. neoformans* (90, 91). In yeast, disruption of Hsp90 affects cell wall integrity and ER stress (92–95). Pyrazolanthrone, also known as SP600125, is a JNK1/2/3 inhibitor used in inflammatory disease, and its interaction with pathogen yeast is unknown (96). Timosaponin AIII induces apoptosis and triggers autophagy in cancer cells (97). The timosaponin AIII cellular response is mediated via inhibition of mTORC1 and induction of ER stress (98); the interaction with pathogen yeast is unknown. CREB (cAMP-response element binding protein) inhibitor (666-15) acts as a bisnaphthamide that disrupts CREB-mediated gene transcription and shows promise as an anti-cancer agent (99). Inhibition of CREB with 666-15 has been shown to impair host antifungal responses and exacerbate hyphal infections in *C. albicans* (100). Polyphyllin I mainly induces apoptosis through the mitochondrial apoptotic pathway as an anticancer agent, leading to accumulation of intracellular ROS (101, 102). Polyphyllin I may have potential as an antifungal because it inhibits the growth of *C. albicans* (103, 104). These findings expand the potential application of antifungal adjuvants targeting ER stress and suggest that interfering with ER homeostasis may be a viable strategy to overcome echinocandin resistance in *C. neoformans*.

Taken together, our results suggest that resistance to echinocandins in *C. neoformans* can be overcome by targeting the ER stress response to influence cell wall remodeling and plasma membrane integrity. While caspofungin alone induced compensatory chitin synthesis and reduced β-glucan production, its combination with tunicamycin inhibited this compensatory response, leading to disruption of cell wall structure and mislocalization of Fks1 (Fig. 9). Combination treatment also impaired capsule attachment, increased cell membrane permeability, and elevated intracellular calcium levels, suggesting that widespread cellular stress affects ER and plasma membrane function (11). Finally, we identified additional ER stress-inducing compounds with potential synergistic effects, highlighting the therapeutic potential of targeting ER homeostasis, ideally with fungal-specific ER targets, to overcome intrinsic echinocandin resistance in *C. neoformans*.

**Fig 9:**
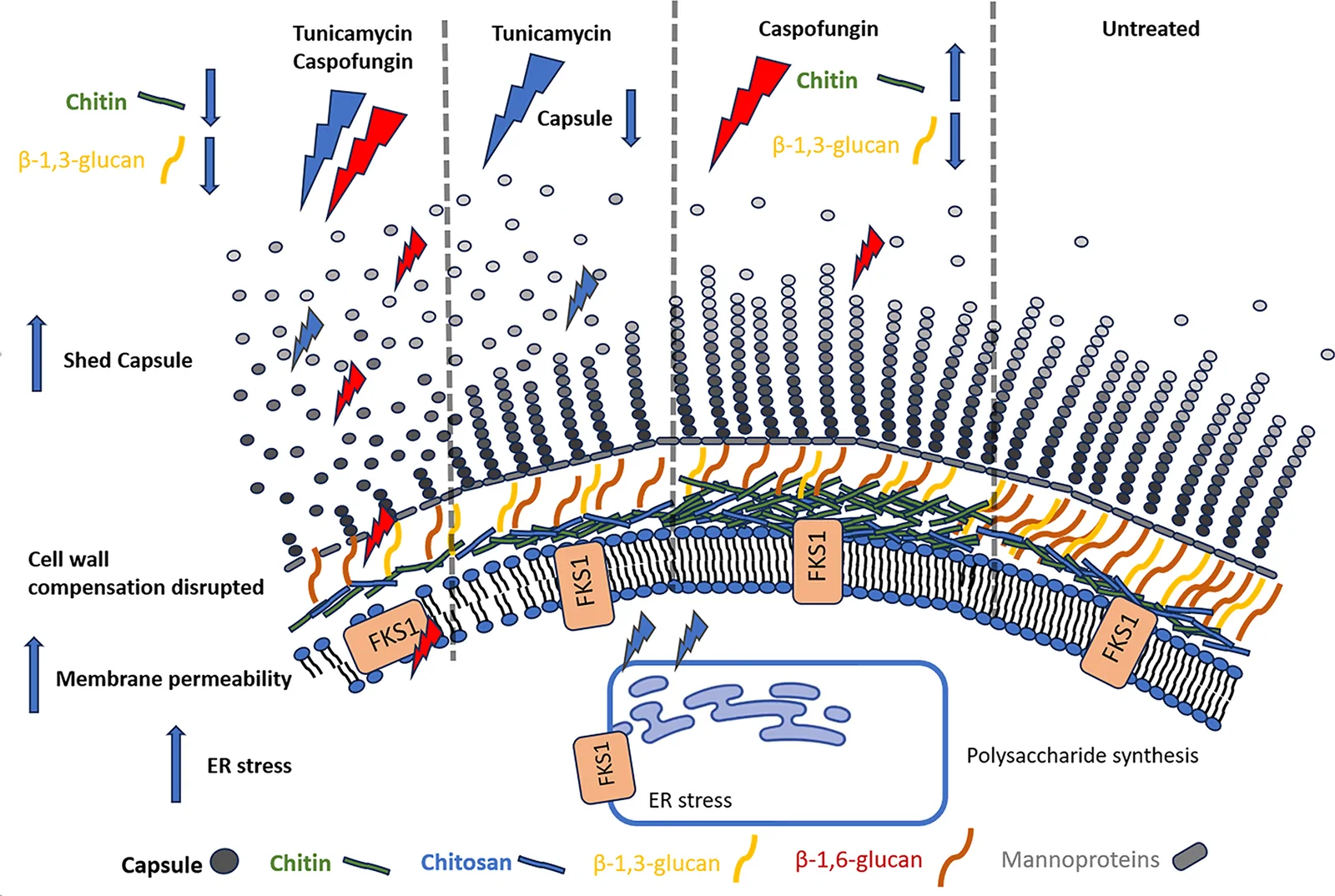
Resistance to echinocandins in *C. neoformans* involves a complex interaction among the ER stress response, cell wall remodeling, and plasma membrane integrity. The polysaccharide capsule, cell wall, and cell membrane are shown for the WT strain under four conditions from left to right: treatment with both caspofungin and tunicamycin; with tunicamycin; with caspofungin; or no treatment. We propose that caspofungin alone induces compensatory increased chitin synthesis and reduced β-glucan production. The combination with tunicamycin is hypothesized to inhibit this compensatory response, leading to severe disruption of cell wall structure and mislocalization of Fks1. The combination of the two inhibitors also impaired capsule attachment and increased cell membrane permeability leading to growth inhibition.

## MATERIALS AND METHODS

### Strains and growth medium

Strain H99 of *C. neoformans* var. *grubii* (serotype A) and derived mutants were used in the experiments. Strains were grown in yeast extract-peptone-dextrose (YPD) (1% yeast extract, 2% peptone, 2% dextrose, 2% agar, broth or agar, pH 7.0) or yeast nitrogen base (YNB; Difco) with amino acids (broth or agar, pH 5.6). Capsule was induced using defined low-iron capsule-inducing medium (LIM) (0.5% glucose, 38 mM L-asparagine, 2.3 mM K_2_HPO_4_, 1.7 mM CaCl_2_·2H_2_O, 0.3 mM MgSO_4_·7H_2_O, 20 mM HEPES, 22 mM NaHCO_3_, 1 mL of 1,000× salt solution (0.005 g/L CuSO_4_·5H_2_O, 2 g/L ZnSO_4_·7H_2_O, 0.01 g/L MnCl_3_·4H_2_O, 0.46 g/L sodium molybdate, 0.057 g/L boric acid), in iron-chelated dH_2_O adjusted to pH 7.4 (with 0.4 mg/L sterile thiamine added), prepared as previously described (105). Dulbecco’s modified Eagle medium (DMEM, Gibco) supplemented with 10% fetal bovine serum, 100 units/mL penicillin, 100 μg/mL streptomycin, and 2 mM L-glutamine at 37°C with 5% CO_2_ was used for propagation of the macrophage-like cell line J774A.1 (ATCC, Manassas, VA). All chemicals were from Sigma-Aldrich (St. Louis, MO, USA) unless otherwise stated.

### Minimal inhibitory concentration (MIC) assays

The MIC assay was performed, as described (17). Tunicamycin was prepared in yeast nitrogen base (YNB) medium and subjected to a twofold serial dilution starting from 4 µg/mL. Caspofungin was similarly serially diluted in YNB medium, beginning at a concentration of 200 µg/mL. The final concentration ranges tested were 7.8  to 4,000 ng/mL for tunicamycin and 0.39  to 200 µg/mL for caspofungin. To determine the lowest MIC₅₀ for caspofungin, we performed a second assay using a concentration range of 0.31  to 25 µg/mL. Cells were grown in YPD overnight in 96-well plates, washed three times with dH_2_O, counted and adjusted to 2 × 10 cells/mL. Cells (10^5^) were added to each well with a corresponding media volume of 200 µL with drugs as indicated. The plates were incubated at 30°C or 37°C for up to 96 h, and the OD_600_ values were recorded every 24 h using a microplate reader (Infinite M200, Tecan). MIC_50_ values were determined by calculating relative growth, defined as the OD₆₀₀ of drug-treated wells divided by the OD_600_ of untreated control wells. The MIC₅₀ was defined as the lowest drug concentration that resulted in a ≥50% reduction in relative growth compared with the untreated control.

### Checkerboard assay

Checkerboard assays were performed to test antifungal drug synergy using the fractional inhibitory concentration index (FIC) approach for combinations of compounds, as previously described (17, 39). Caspofungin was diluted for a final concentration range between 1.5625 and 100 μg/mL, tunicamycin was diluted for a final concentration range of 7.8  to 4,000 ng/mL. The other ER inhibitors were diluted to the following concentration ranges: RA190 0.625 to 50 µM, honokiol 0.625 to 50 µM, HSP990 0.65 to 20 µM, and pyrazolanthrone 0.65 to 20 µM. Timosaponin AIII 0.65 to 20 µM, CREB inhibitor 0.65 to 20 µM, and polyphyllin I 0.65 to 20 µM. Assays were incubated at 30 and 37°C. FIC index values for a combination of compounds A and B were calculated with the following formula:


$$FIC=\frac{MICA\;in\;combination}{MICA\;alone\;}+\frac{MICB\;in\;combination}{MICB\;alone}.$$

An FIC index value of less than 1.0 is considered synergistic (with <0.5 considered strongly synergistic), 1.0 is considered additive, and a value between 1.0 and 2.0 is considered autonomous (15, 17, 39).

### Assessment of capsule formation and shedding

Differential interference contrast (DIC) microscopy was employed to examine capsule formation. After growth in YPD at 30°C overnight, cells were washed three times with LIM, and transferred into 2 mL LIM supplemented with 10 µg/mL caspofungin, 125 ng/mL tunicamycin, or both caspofungin and tunicamycin at the same concentrations. The additional ER inhibitors including RA190, honokiol, HSP990, and pyrazolanthrone (all at 5 µM) were tested with 10 µg/mL caspofungin. After incubation at 30°C or 37°C for 48 h, cells were imaged with India ink staining using a Zeiss Plan-Apochromat 100×/1.46 oil lens on a Zeiss Axioplan 2 microscope. To assess shedding, cells were grown in YPD overnight, washed three times with LIM, transferred to 3 mL LIM, and incubated for 48 h at 30°C or 37°C. Each culture was normalized based on cell OD_600_, the supernatants were denatured at 70°C for 15 min, subjected to electrophoresis on a 1% agarose gel, and blotted onto a nylon membrane (GE Healthcare, Boston, MA, USA). Capsule material on the membrane was detected by incubation with a 1:5,000 dilution of the 18B7 monoclonal antibody, followed by incubation with a 1:5,000 dilution of anti-mouse HRP (Bio-Rad). The polysaccharide was then visualized by chemiluminescence (GE Healthcare, Boston, MA, USA).

### Transmission electron microscopy

After overnight growth in YPD, cells were transferred to YNB with or without drug treatment and normalized to an OD_600_ of 2. The cells were then incubated for 48 h at 30°C, washed three times in sterile dH_2_O, and fixed in 2.5% glutaraldehyde in 0.1 M sodium cacodylate, pH 6.9, for 1 h at room temperature (RT). Cells were then washed three times with 0.1 M sodium cacodylate buffer to remove the fixative solution. The fixed cells were separated in 3% low-temperature gelling agarose and post-fixed with 1% OsO_4_, 0.8% K_4_[Fe(CN)_6_], 5 mM CaCl_2_ in 0.1 M sodium cacodylate at pH 7.4 for 10 min. After three subsequent washes with dH_2_O and incubation in 1% thiocarbohydrazide (TCH) in dH_2_O for 5 min, the cells were again washed three times in dH_2_O and post-fixed for 2 min. The cells were then dehydrated by sequential washes with a graded concentration series of acetone into 100% ethanol. Cells were embedded in Spurr’s resin and a Leica Ultramicrotome UCT was used to cut 70-nm sections. Sections were stained with 2% uranyl acetate (UA) for 12 min, followed by Reynolds’ lead citrate for 6 min. A FEI Tecnai Spirit 120-kV transmission electron microscope operated at an accelerating voltage of 80 kV was used for image collection, and images were acquired with a DVC1500M side-mounted camera controlled by AMT software.

### RNA extraction and RT-qPCR

After overnight growth in YPD media to log phase, cells were washed twice in YNB, and 2 × 10^7^ cells were transferred to YNB (2% glucose) at 30°C or 37°C with or without the following treatments: 10 µg/mL caspofungin, 125 ng/mL tunicamycin, or both caspofungin and tunicamycin. Cells were harvested following 2 and 6 h of culture, frozen in liquid nitrogen, and stored at −80°C. RNA was extracted from cells with lysis buffer (2% SDS, 68 mM tri-sodium citrate, 132 mM citric acid, 10 mM EDTA, pH 3.5) and extraction buffer (4 M sodium chloride, 17 mM tri-sodium citrate, 33 mM citric acid, pH 3.5). Turbo Dnase (Ambion, Austin, TX) was used for DNase treatment according to the manufacturer’s instructions, and cDNA synthesis was performed using the Verso cDNA reverse transcription kit (Thermo Fisher Scientific, Waltham, MA) with oligo (dT). The Green-2-Go qPCR Mastermix with the primers listed in Table S3 was employed for quantitative reverse transcription PCR (qPCR). The transcripts for glyceraldehyde phosphate dehydrogenase (*GAPDH*) and actin (*ACT1*) were used for normalization. The following genes were analyzed: UPR related functions (*IRE1, HXL1_1, HXL1_2*), capsule formation (*CAP60, CAP64*), chitin synthesis (*CHS1*, *CHS2*, *CHS3*, *CHS4*, *CHS5*, *CHS6*, *CHS7*, *CHS8*), chitosan formation (*CDA1*, *CDA2*, *CDA3*), α-1,3-glucan synthesis (*AGS1*), β-1,3-glucan synthesis (*FKS1*), and β-1,6-glucan synthesis (*KRE6* and *SKN1*). The 2^−ΔΔCt^ method was used to quantify relative transcript levels.

### Microscopy and flow cytometry

Cells were grown overnight in YPD, and cultures were adjusted to OD _600_= 1 before transfer into four treatment conditions: fresh YNB, YNB with 10 µg/mL caspofungin, with 125 ng/mL tunicamycin, or with both caspofungin and tunicamycin. Cultures (3 mL) were incubated at 30°C or 37°C for 48 h. The cultures were then diluted, and an aliquot of cells (OD _600_= 1) from each treatment was collected for staining. For microscopy, cells expressing Fks1-GFP were fixed with 2% paraformaldehyde (PFA) at RT for 20 min, then stained with 5 µM/mL DAPI (4′,6-diamidino-2-phenylindole) for nuclei at RT in the dark for 30 min. Cells were imaged using a Zeiss Plan-Apochromat 100×/1.46 oil lens on a Zeiss Axioplan 2 microscope, and cell size was measured by ImageJ.

Chitin was detected using 100 µg/mL calcofluor white (CFW), incubated for 5 min at RT, followed by flow cytometry using the Pacific Blue channel (PB450, 405/450 nm). β-glucan was detected using 0.05% aniline blue, incubated for 10 min in the dark at RT, and analyzed using the Pacific Blue channel (PB450, 405/450 nm). Chitin oligomers of N-acetylglucosamine were detected using 10 µg/mL fluorescein-conjugated wheat germ agglutinin (WGA), incubated for 30 min in the dark at RT, followed by flow cytometry using the FITC channel (488/530 nm). Membrane permeability and cell viability were detected by 5 μg/mL propidium iodide, incubated in the dark at 37°C for 30 min, and the PE channel (493/636 nm) was used for flow cytometry. For membrane permeability, cells were incubated in YNB for 3 h at 30°C with different SDS concentrations (0.005% and 0.01%). For intracellular calcium levels, cells were incubated in YNB for 3 h at 30°C with different CaCl_22_ concentrations (100 and 200 mM). Calcium levels were measured with 5-μM calcium indicator Cal Green 1 AM (Calcium Green-1 AM) in the dark at 37°C for 30 min, followed by flow cytometry using the FITC channel (488/530 nm). Flow cytometry was performed using a CytoFLEX (Beckman Coulter), and data were analyzed using FlowJo v10.

### Spot assay for drug sensitivity

YNB agar was amended with the following reagents for drug resistance testing: 125 ng/mL tunicamycin; 10 µg/mL caspofungin; 0.005% SDS (plasma membrane stress); 1.5 M KCl, 1.5 M NaCl, 1.5 M sorbitol (osmotic stress); 1 mg/mL calcofluor white (CFW for cell wall stress), and 0.5 mg/mL caffeine (cell wall integrity). Briefly, cells were grown in YPD overnight, washed three times with sterile YNB, and OD_600_ = 1 cells were transferred into 3 mL with four conditions: fresh YNB, YNB with 10 µg/mL caspofungin, with 125 ng/mL tunicamycin, or with both caspofungin and tunicamycin. After incubating at 30°C for 48 h. Cells were counted and adjusted to 2 × 10^7^ cells/mL in PBS. Serial dilutions (1/10) were performed and 5 µL (3 µL for SDS plates) of each dilution was spotted onto agar medium (10^5^ cells for the first spot). Plates were incubated at 30°C or 37°C for 3 days before imaging.

### Extracellular acid phosphatase assay

Cells were grown overnight in YPD, and transferred into four 3-mL treatment conditions: fresh YNB, YNB with 10 µg/mL caspofungin, YNB with 125 ng/mL tunicamycin, YNB containing both caspofungin and tunicamycin for 48 h at 30°C. The cells were then washed with distilled water and resuspended in MM-250 mM KH_2_PO_4_ or MM-0 Pi medium at an OD_600_ of 2 in 3 mL. The cultures were incubated at 30°C for 3 h. After incubation, cells were centrifuged for 5 min at maximum speed. Supernatants (50 µL) of each sample were assayed for acid phosphatase activity using the Acid Phosphatase Assay Kit from Sigma Aldrich (MAK446). Reaction mixtures were incubated at 37°C and stopped at 10, 30, or 60 min by adding 200 µL of 0.5 N NaOH. The extent of 4-nitrophenyl phosphate hydrolysis was measured spectrophotometrically at 405 nm. The assay was performed in triplicate.

### Screening a TargetMol endoplasmic reticulum stress compound library

A screen was performed in 96-well plates to identify potential synergistic interactions between caspofungin and compounds causing endoplasmic reticulum (ER) stress (TargetMol: https://www.targetmol.com/compound-library/endoplasmic_reticulum_stress_compound_library). Cells were grown in YPD overnight, washed three times with YNB medium, and resuspended to a final concentration of 2 × 10⁷ cells/mL. A total of 5 µL of cell suspension (1 × 10⁵ cells) was added to each well containing 195 µL of YNB medium supplemented with 10 µM of a library compound in the presence or absence of caspofungin (10 µg/mL or 20 µg/mL). The final volume per well was 200 µL. Control wells included cells incubated in YNB alone or with 10 µg/mL caspofungin. Plates were incubated at 30°C with shaking (180 rpm), and OD_600_ was measured every 24 h for up to 72 h using a Tecan Infinite M200 microplate reader. Each condition was tested in three biological replicates across two independent experiments. RA190, honokiol, HSP990 20 to 0.65 µM, pyrazolanthrone, timosaponin, CREB inhibitor, and polyphyllin I were found to inhibit wild-type (WT) growth; their synergistic effect with caspofungin was further analyzed using a checkerboard assay.

### Phagocytosis assay

Intracellular proliferation assays were performed using the murine macrophage-like cell line J774A.1 (ATCC, Manassas, VA), as previously described (106, 107). *Cryptococcus* cells were grown in YPD overnight at 200 rpm, 30°C, and transferred into YNB with or without drug treatment (10 µg/mL caspofungin, 125 ng/mL tunicamycin, or both drugs) for 48 h. J774A.1 cells were seeded to 1 × 10^5^ cells/mL per well (three wells for each treatment, 1 mL in each well) in a 24-well plate, incubated at 37°C, 5% CO_2_ for 24 h prior to infection. Half of the fungal cells were opsonized with 18B7 anti-capsule antibody at a final concentration of 10 μg/mL, and half were not, as indicated. J774A.1 cells were activated with 150 ng/mL of phorbol 12-myristate 13-acetate (PMA; Sigma-Aldrich, St. Louis, MO) in serum-free DMEM (sfDMEM) with incubation at 37°C, 5% CO_2_ for 1 h. Macrophages were then incubated with the fungal cells for 2 h at a multiplicity of infection 1:2 (macrophage:yeast) at 37°C with 5% CO_2_. For intracellular CFUs, external yeast cells were washed off with sterile PBS (4 or 5 washes) after 2 h of incubation, and half of the macrophages were lysed with sterile distilled water to collect intracellular fungal cells (labeled as 2 h timepoint). After 24 h of incubation, sterile, ice‐cold distilled H_2_O was applied to lyse the rest of the macrophages (labeled as the 24-h timepoint). Fungal growth was measured by counting CFUs after 2 days of growth on YPD.

### Statistical analyses

Image J was used to measure cell diameter, cell wall size, and capsule thickness. All assays, including spot assays, capsule measurements, growth curves, flow cytometry, phagocytosis, microscopy, and qPCR, were performed three times. Means and standard deviations are shown, and statistical significance was determined by one-way ANOVA and Tukey’s multiple comparisons in GraphPad Prism 8.0 (GraphPad Software).

## SUPPLEMENTAL MATERIAL

Supplemental Figures msphere.00451-26-s0001.pdf Fig. S1–S9.

Table S1 msphere.00451-26-s0002.docx FIC of caspofungin with tunicamycin at pH 7.

Table S2 msphere.00451-26-s0003.xlsx TargetMol ER stress library screening data.

Table S3 msphere.00451-26-s0004.xlsx Primer sequences for qPCR.

ASM does not own the copyrights to Supplemental Material that may be linked to, or accessed through, an article. The authors have granted ASM a non-exclusive, world-wide license to publish the Supplemental Material files. Please contact the corresponding author directly for reuse.
