# Supplementary material for: Endoplasmic reticulum stress reduces caspofungin resistance in Cryptococcus neoformans
Source: mSphere. 2026 Sep 4;11(9):e00451-26. doi: 10.1128/msphere.00451-26 (PMC13621870; doi:10.1128/msphere.00451-26)
Supplement: Supplementary file 1 [file msphere.00451-26-s0001.pdf]

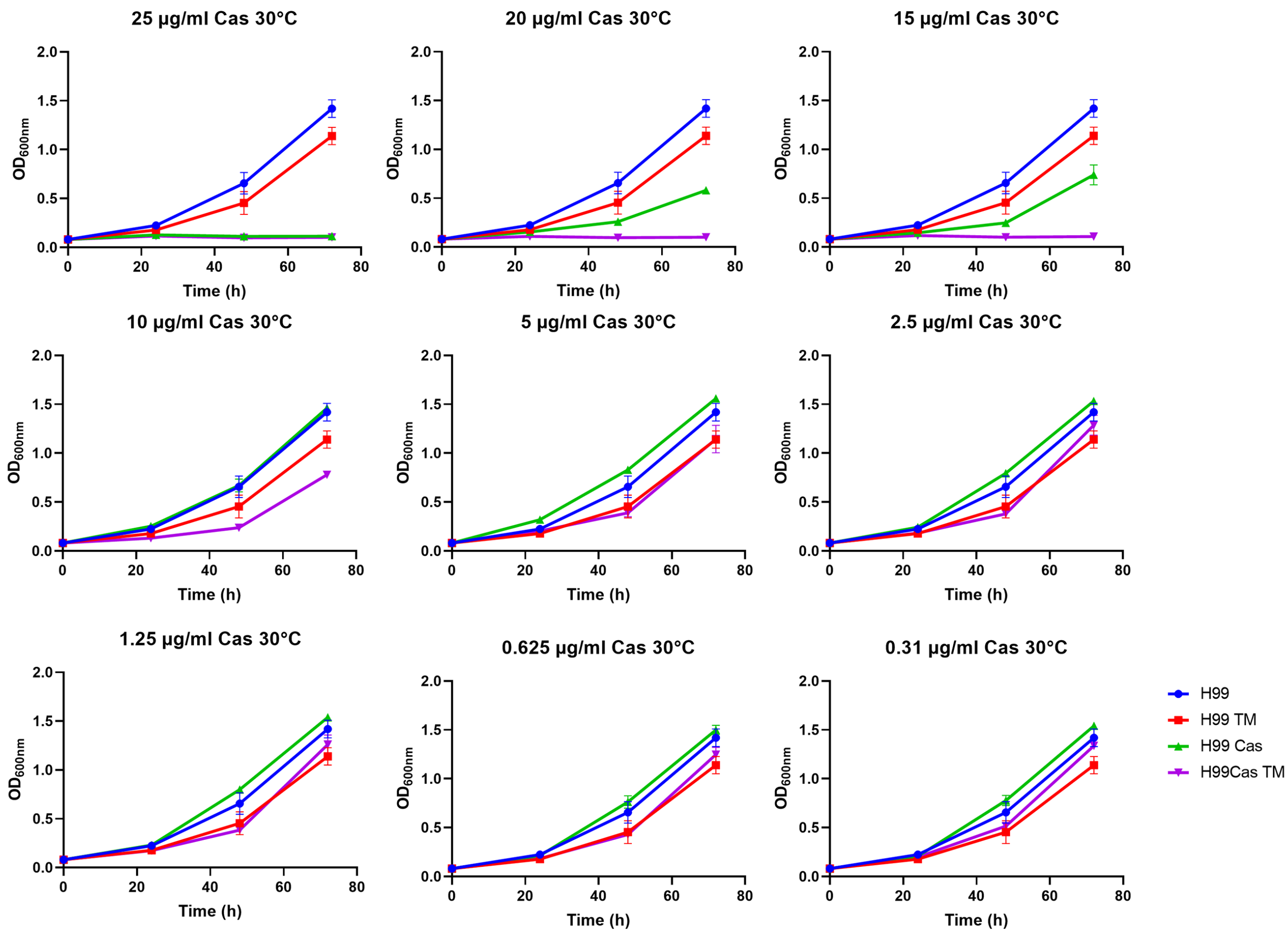

Figure S1. Growth of *C. neoformans* WT strain (H99) in the presence of caspofungin and tunicamycin.

Cells of the WT H99 strain were grown overnight in YPD at 30°C, washed, and transferred into YNB medium in 96-well plates for growth assays. Caspofungin was tested in a concentration range of 25–0.31 µg/mL, and tunicamycin was used at a fixed concentration of 125 ng/mL. OD_{600} was measured every 24 h. Each condition was tested in three independent experiments with three biological replicates and the error bars indicate the standard deviation for each measurement. In the graph, blue represents H99 grown in YNB without drug treatment, red indicates H99 with 125 ng/mL tunicamycin, green represents H99 treated with caspofungin alone, and purple indicates H99 treated with both caspofungin and tunicamycin.

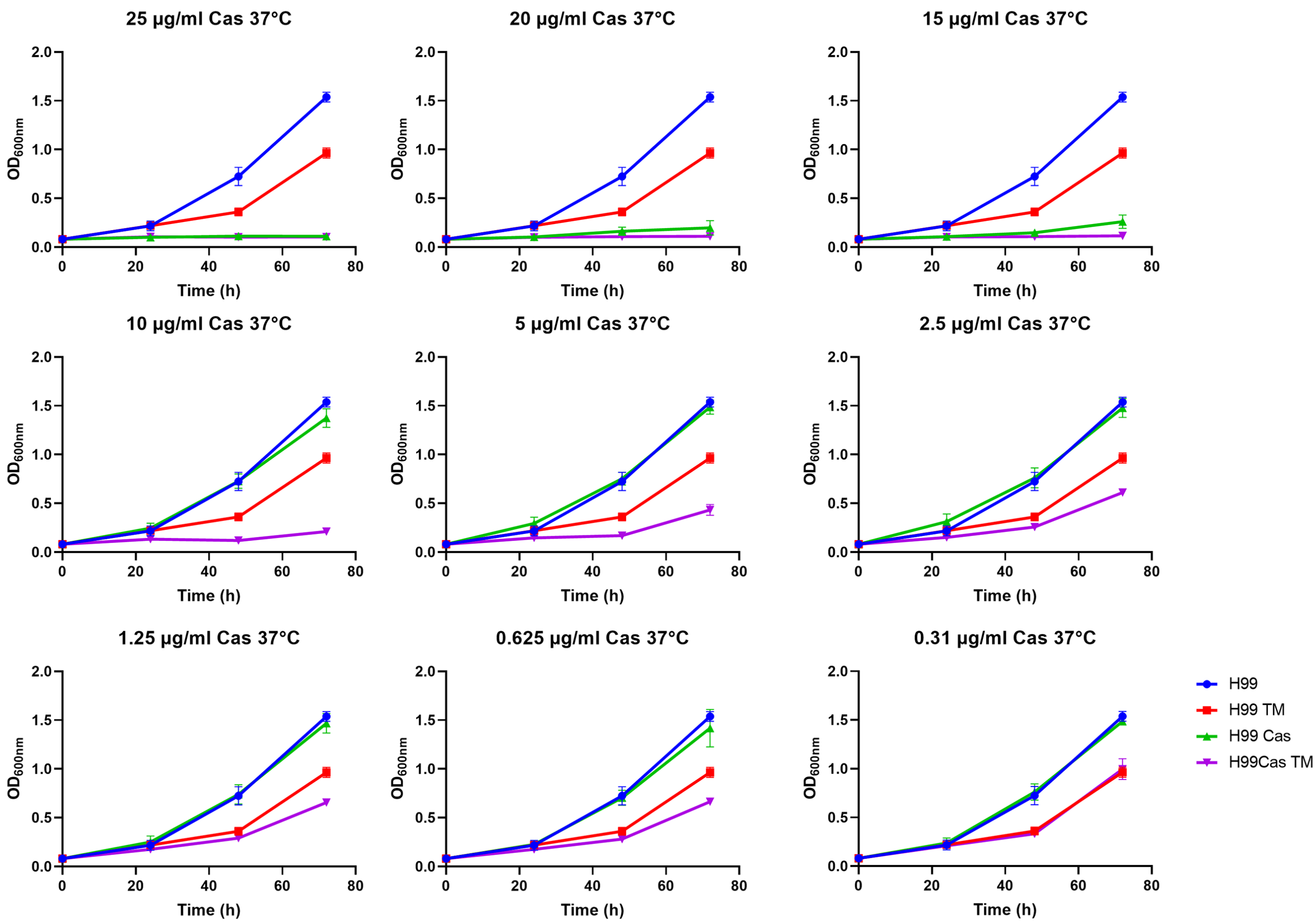

Figure S2. Growth of *C. neoformans* WT strain (H99) in the presence of caspofungin and tunicamycin.

Cells of the WT H99 strain were grown overnight in YPD at 37°C, washed, and transferred into YNB medium in 96-well plates for growth assays. Caspofungin was tested in a concentration range of 25–0.31 µg/mL, and tunicamycin was used at a fixed concentration of 125 ng/mL. OD_{600} was measured every 24 h. Each condition was tested in three independent experiments with three biological replicates and the error bars indicate the standard deviation for each measurement. In the graph, blue represents H99 grown in YNB without drug treatment, red indicates H99 with 125 ng/mL tunicamycin, green represents H99 treated with caspofungin alone, and purple indicates H99 treated with both caspofungin and tunicamycin.

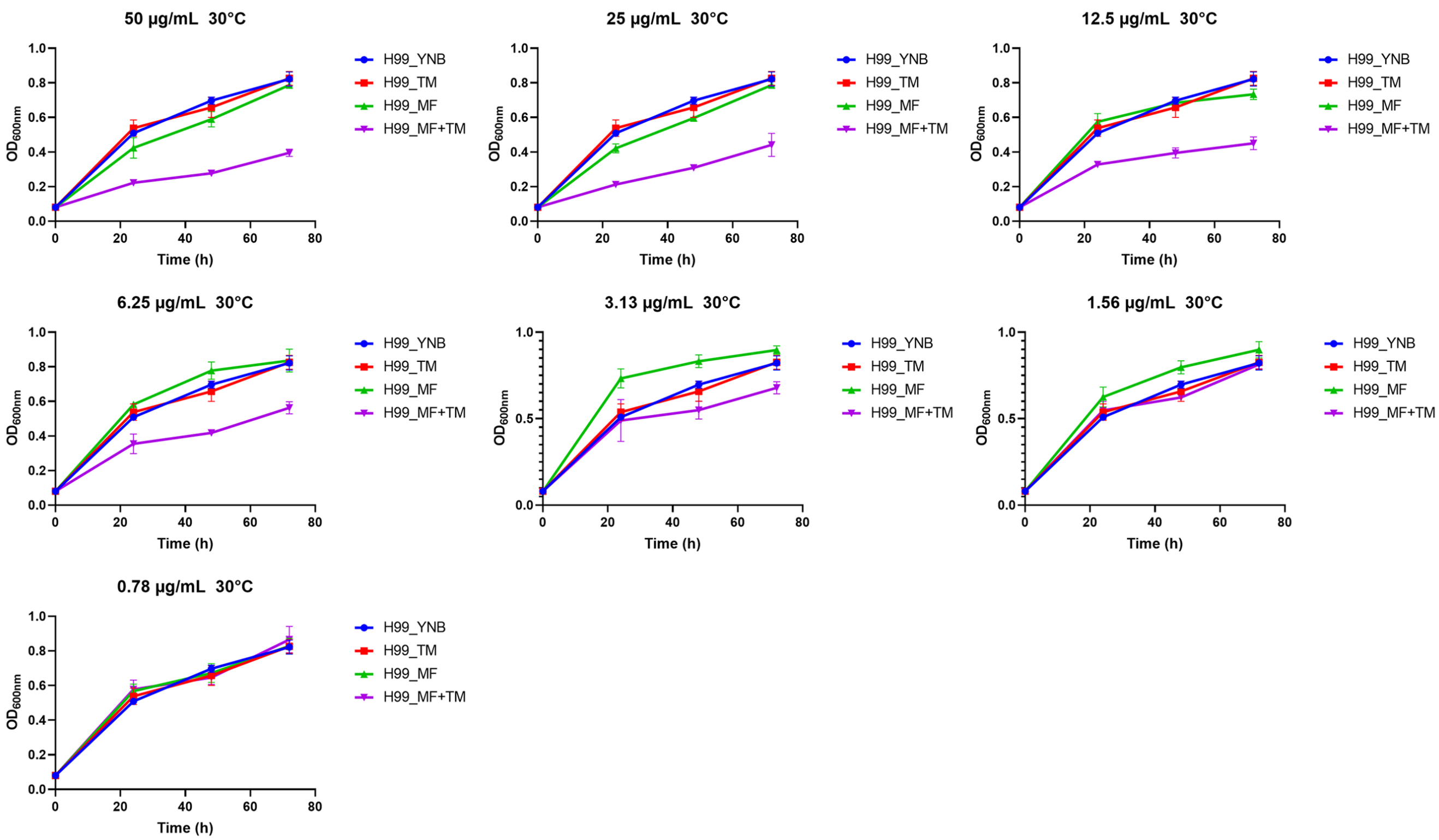

Figure S3. Growth of *C. neoformans* WT strain (H99) in the presence of micafungin and tunicamycin.

Cells of the WT H99 strain were grown overnight in YPD at 30°C, washed, and transferred into YNB medium in 96-well plates for growth assays. Caspofungin was tested in a concentration range of 50–0.78 µg/mL, and tunicamycin was used at a fixed concentration of 125 ng/mL. OD_{600} was measured every 24 h. Each condition was tested in three independent experiments with three biological replicates and the error bars indicate the standard deviation for each measurement. In the graph, blue represents H99 grown in YNB without drug treatment, red indicates H99 with 125 ng/mL tunicamycin, green represents H99 treated with caspofungin alone, and purple indicates H99 treated with both caspofungin and tunicamycin.

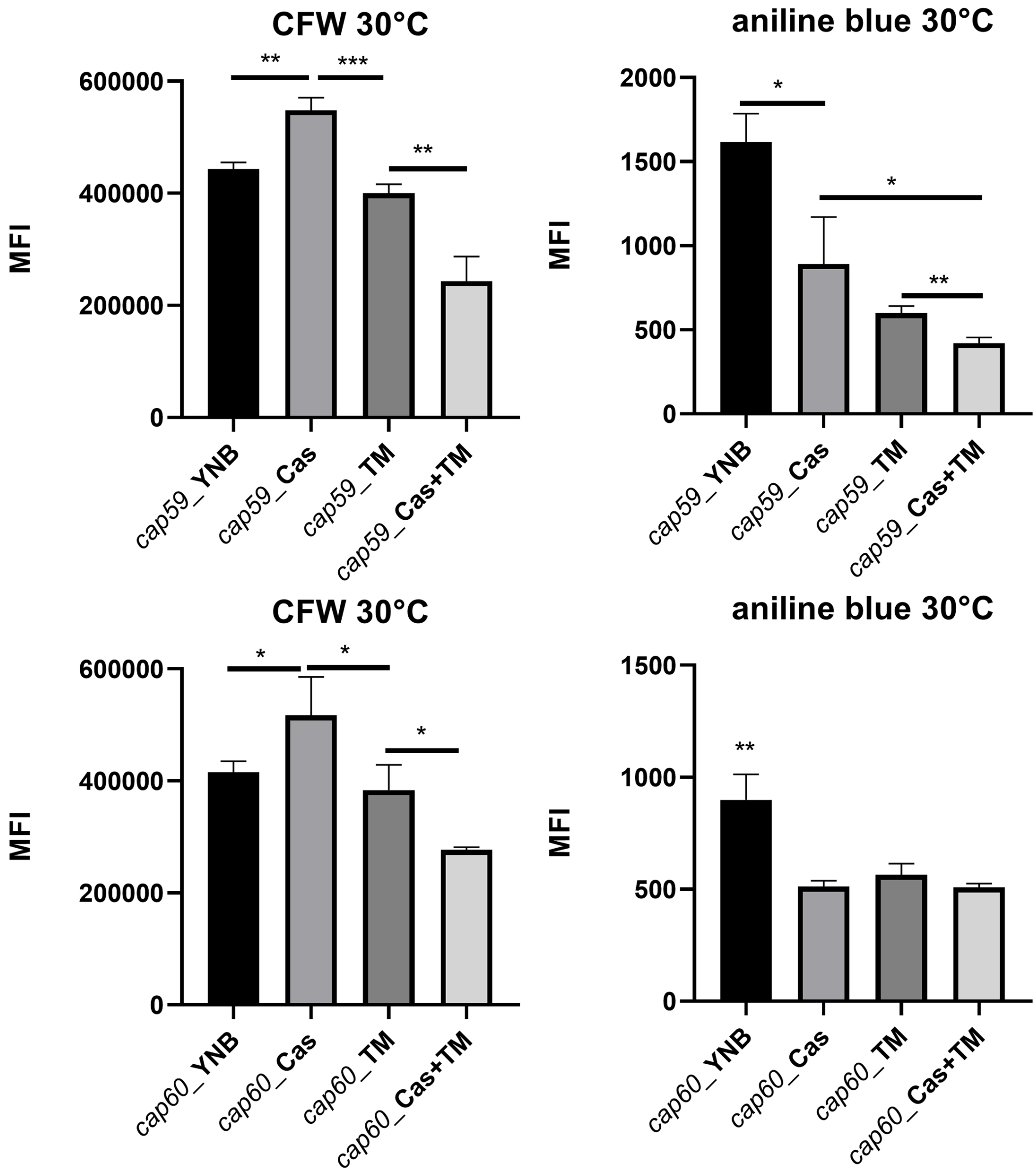

Figure S4. Cell Wall Defects Induced by Combined Caspofungin and Tunicamycin Treatment in *cap59* and *cap60* mutants.

Levels of chitin (CFW) and β-glucan (aniline blue) determined by flow cytometry with Pacific Blue (PB450, 405/450 nm). Cells were pre-grown in YPD and then transferred into minimal YNB medium without or with the drugs (10 µg/mL caspofungin, 125 ng/mL tunicamycin) for 24 h at 30°C. ΔMFI = change in mean fluorescence intensity. Error bars represent standard deviation. Statistical significance was determined using one-way ANOVA with Tukey's multiple comparisons in GraphPad Prism 8.0 (GraphPad Software) (*p<0.05 **p<0.01, ***p<0.001 and ****p<0.0001).

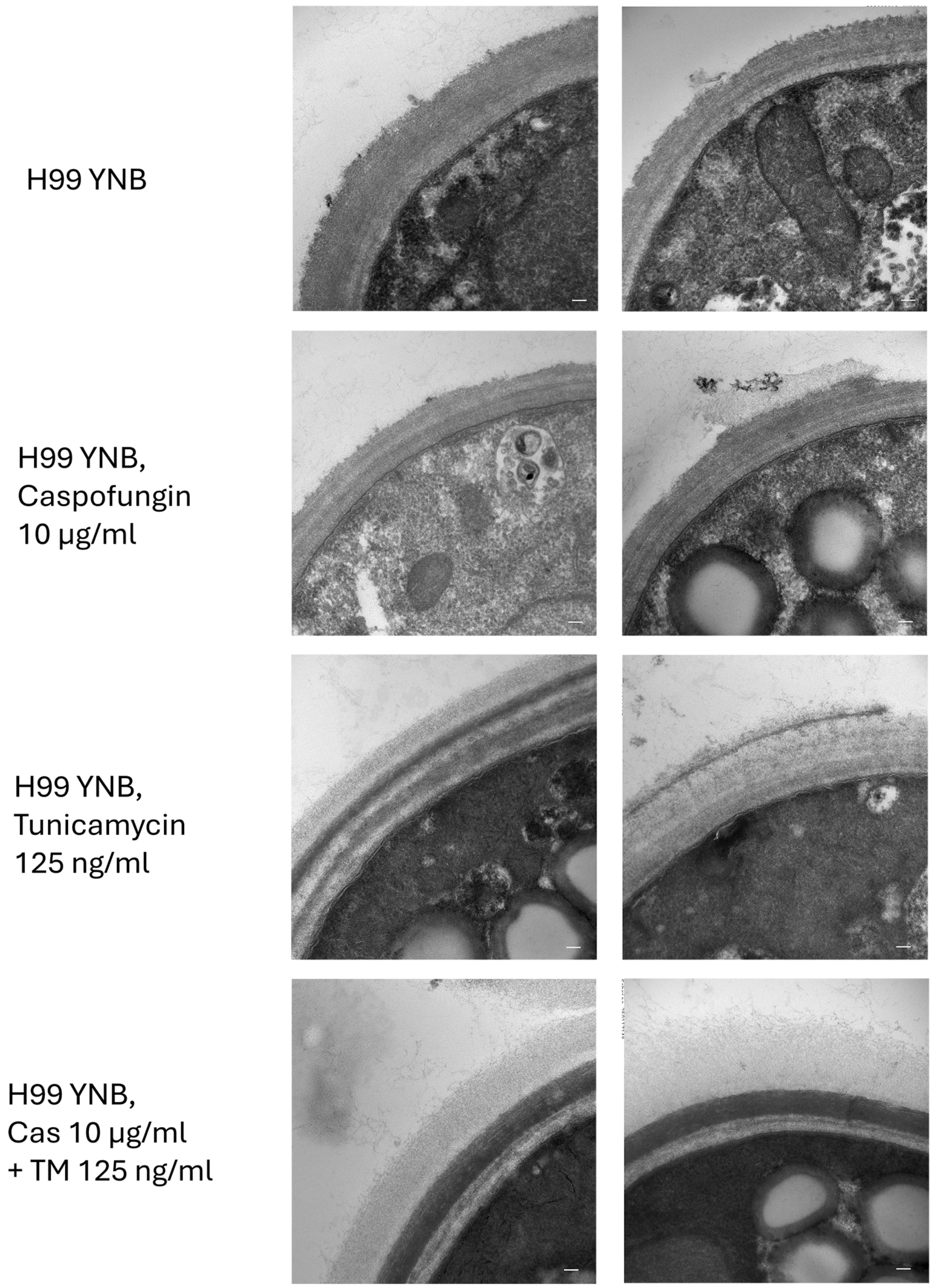

Figure S5. TEM showed Cell Wall Defects Induced by Combined Caspofungin and Tunicamycin.

Additional representative images of the cell walls obtained by transmission electron microscopy (TEM) from Figure 2, scale bar = 100 nm. Cells were grown overnight in YPD, transferred to YNB with or without drug treatment for 48 h at 30°C.

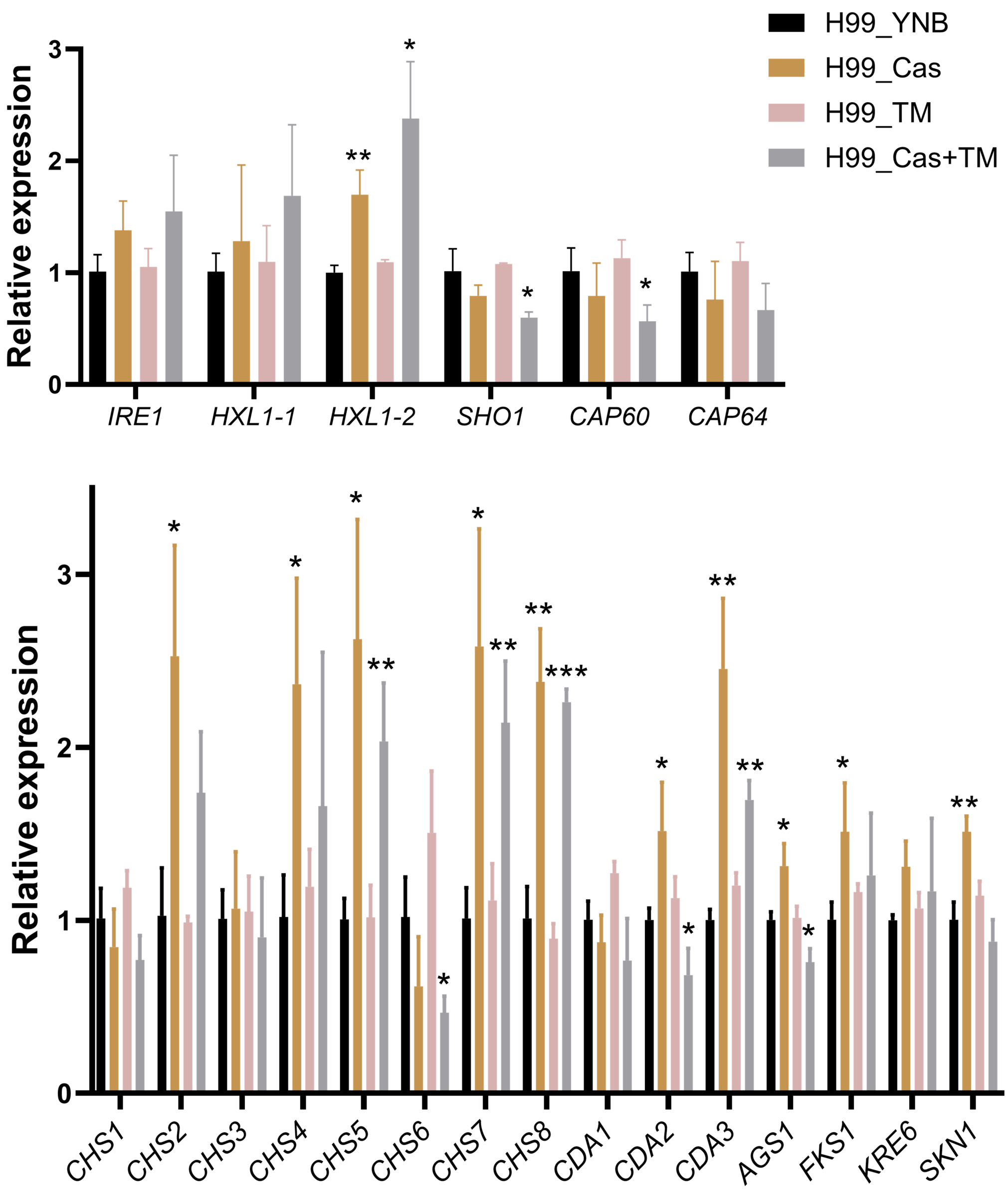

Figure S6. Cell wall gene expression is altered during caspofungin and tunicamycin treatment

qPCR analysis of the transcript levels for genes related to the UPR, capsule and cell wall synthesis upon growth in drug treatment conditions for 2 h at 37°C. The relative expression levels were compared between YNB and other conditions (10 µg/mL caspofungin,125 ng/mL tunicamycin, combine caspofungin and tunicamycin), using the housekeeping gene *GAPDH* as control. The assays were performed twice with three biological replicates each. The mean and standard deviation of the measurements are shown. Statistical significance was determined by performing with one-way ANOVA and Tukey's multiple comparisons in GraphPad Prism 8.0 (GraphPad Software). (*p<0.05 **p<0.01 and ***p<0.001: compared with the WT strain in YNB alone).

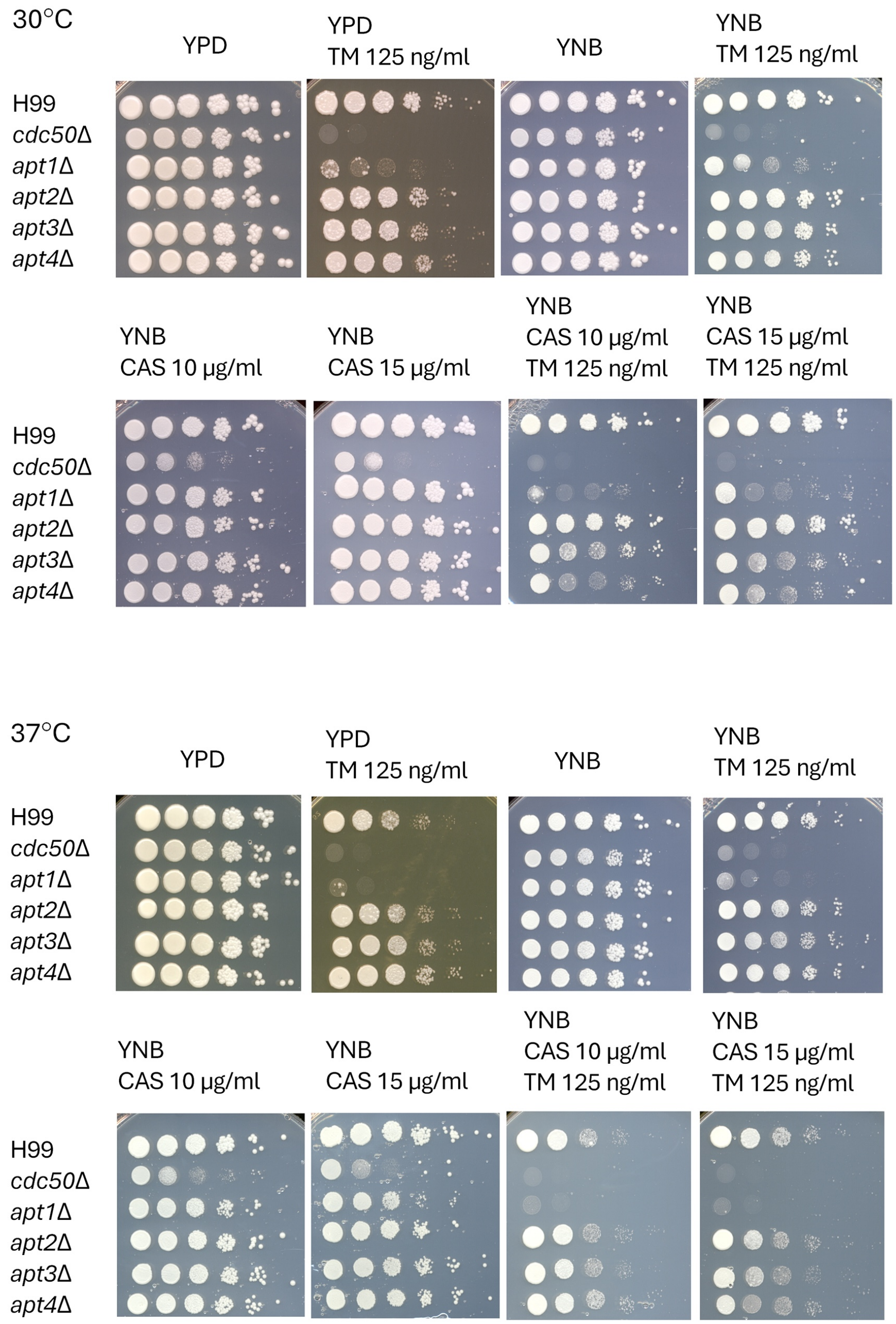

Figure S7. *cdc50* and *apt1* mutants showed increased sensitivity to combined tunicamycin and caspofungin.

YNB agar was added with the following concentrations of reagents for drug resistance testing: 125 ng/mL tunicamycin; 10-15 µg/mL caspofungin or both. WT and mutant cells were grown in YPD overnight, washed three times with sterile YNB, OD600=1 cells were transferred into four conditions with 3mL: fresh YNB, YNB with 10 µg/mL caspofungin, YNB with 125 ng/mL tunicamycin or YNB with both caspofungin and tunicamycin. After incubating at 30°C for 48 h. Cells were counted and adjusted to $2x10^7$ cells/mL in PBS. Serial dilutions (1:10) were performed and 5 µl (3 µl for SDS plates) of each dilution was spotted onto agar medium (10^5 cells for first spot). Plates were incubated at 30 °C or 37 °C for 3 d before imaging.

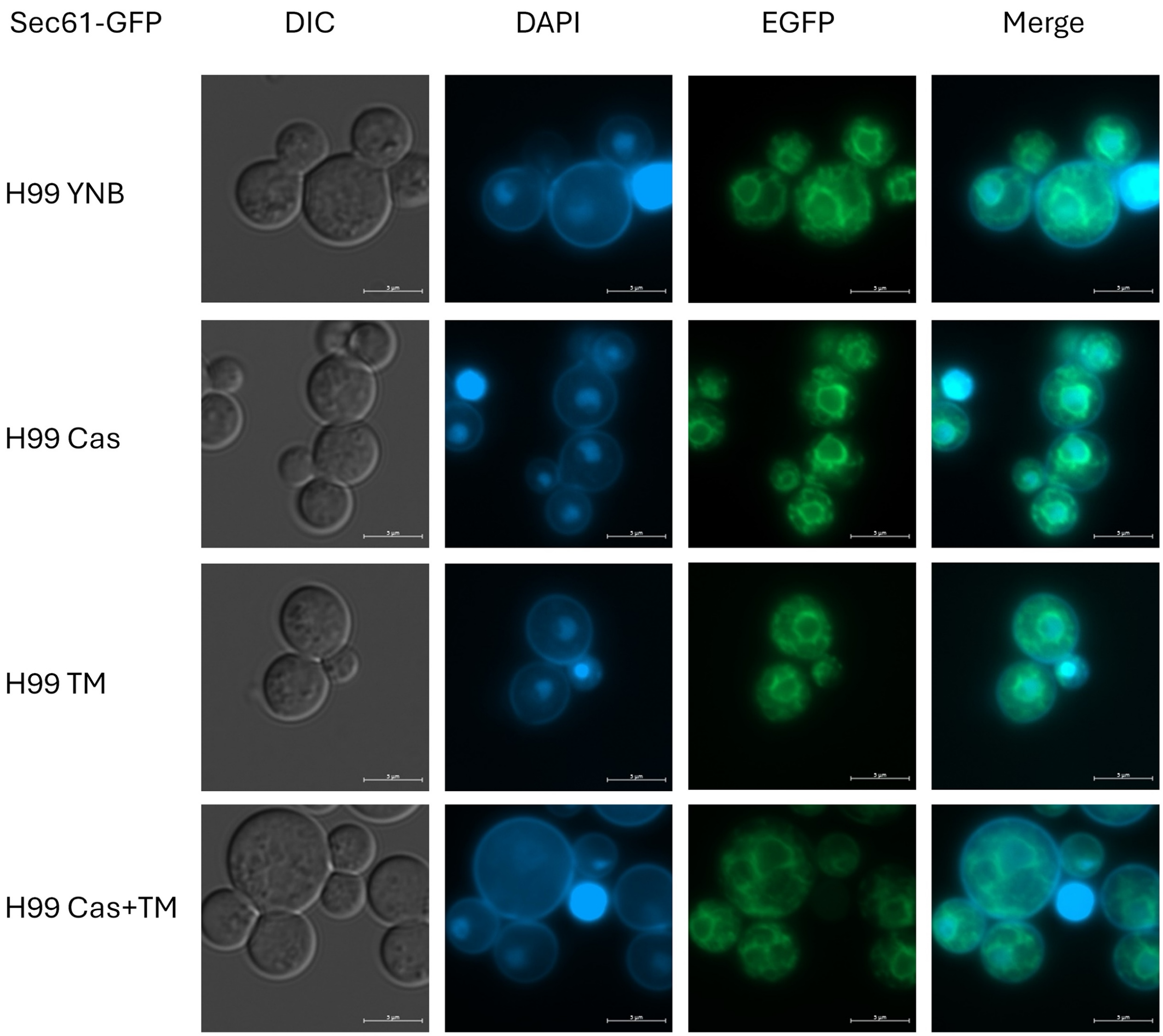

Figure S8. Sec61-GFP localization is not altered during combined treatment.

Sec61-GFP Cells were grown overnight in YPD and then transferred into four 3 mL treatment conditions: fresh YNB, YNB with 10 µg/mL caspofungin, YNB with 125 ng/mL tunicamycin, YNB containing both caspofungin and tunicamycin for 24 h at 30 °C. Cells at OD_{600} = 1 were collected and fixed with 2% paraformaldehyde (PFA) at RT for 20 minutes, then stained with 5 µM DAPI (4′,6-diamidino-2-phenylindole) for nuclei at RT in the dark for 30 min. 5 µl cells were imaged using a Zeiss Plan-Apochromat 100×/1.46 oil lens on a Zeiss Axioplan 2 microscope (scale bar = 5µm).

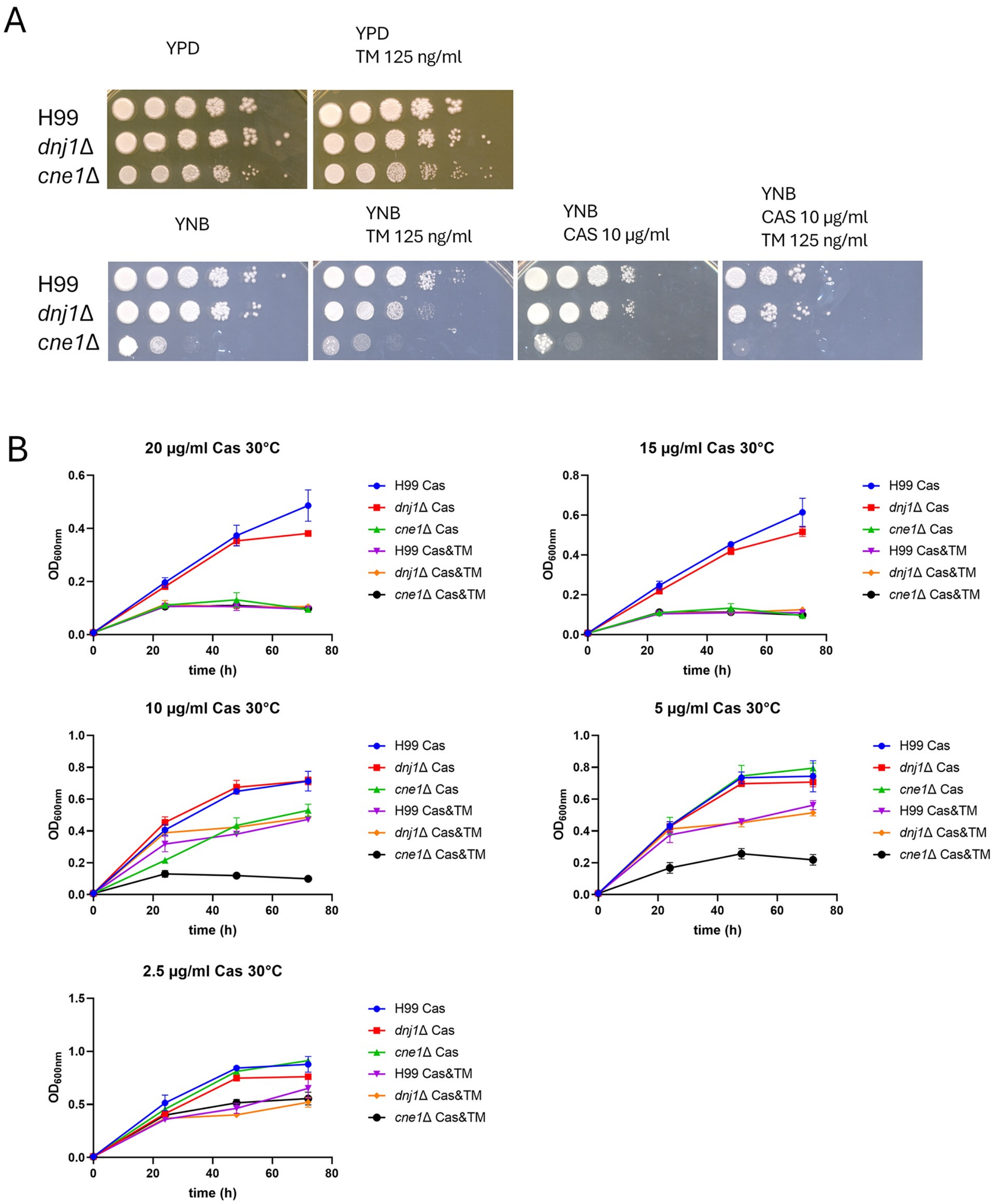

Figure S9. The *cne1* mutant has increased sensitivity to tunicamycin and caspofungin.

(A) Spot assays to measure growth in YNB agar with 125 ng/mL tunicamycin, 10 µg/mL caspofungin or both drugs. WT and mutant cells were grown in YPD overnight, and cells were counted and adjusted to 2×10^7 cells/mL in PBS. Serial dilutions (1:10) were performed and 5 µl of each dilution was spotted onto agar medium (10^5 cells for first spot). Plates were incubated at 30°C for 3 d before imaging. (B) Cells were grown overnight in YPD at 30°C, washed, and transferred into YNB medium in 96-well plates at 30°C for growth assays. Caspofungin was tested in a concentration range of 20–2.5 µg/mL, and tunicamycin was used at a fixed concentration of 125 ng/mL. OD_{600} was measured every 24 h. Each condition was tested in three independent experiments with three biological replicates.
