# Supplementary material for: Endoplasmic reticulum stress reduces caspofungin resistance in Cryptococcus neoformans
Source: mSphere. 2026 Sep 4;11(9):e00451-26. doi: 10.1128/msphere.00451-26 (PMC13621870; doi:10.1128/msphere.00451-26)
Supplement: Supplementary file 2 [file msphere.00451-26-s0002.docx]

Table S1 Minimal inhibitory concentration (MIC) and Fractional Inhibitory concentration Index (FIC) of Caspofungin with Tunicamycin at pH 7.

| Strain H99 | | MIC_50_ (30^o^C) | | | FIC |
| --- | --- | --- | --- | --- | --- |
|  | Alone | | Combined | |  |
|  | Tunicamycin | Caspofungin | Tunicamycin | Caspofungin |  |
|  | 500 ng/ml | 25 μg/ml | 125 ng/ml | 15 μg/ml | 0.85 |
|  | |  | |  |  |
| Strain H99 | | MIC_50_ (37^o^C) | | FIC |  |
|  | Alone | | Combined | |  |
|  | Tunicamycin | Caspofungin | Tunicamycin | Caspofungin |  |
|  | 500 ng/ml | 15 μg/ml | 125 ng/ml | 0.625 μg/ml | 0.29 |
